# A feedback-driven brain organoid platform enables automated maintenance and high-resolution neural activity monitoring

**DOI:** 10.1016/j.iot.2025.101671

**Published:** 2025-07-11

**Authors:** Kateryna Voitiuk, Spencer T. Seiler, Mirella Pessoa de Melo, Jinghui Geng, Tjitse van der Molen, Sebastian Hernandez, Hunter E. Schweiger, Jess L. Sevetson, David F. Parks, Ash Robbins, Sebastian Torres-Montoya, Drew Ehrlich, Matthew A.T. Elliott, Tal Sharf, David Haussler, Mohammed A. Mostajo-Radji, Sofie R. Salama, Mircea Teodorescu

**Affiliations:** a Genomics Institute, University of California Santa Cruz, Santa Cruz, 95064, CA, USA; b Department of Biomolecular Engineering, University of California Santa Cruz, Santa Cruz, 95064, CA, USA; c Department of Electrical and Computer Engineering, University of California Santa Cruz, Santa Cruz, 95064, CA, USA; d Neuroscience Research Institute, University of California Santa Barbara, Santa Barbara, 93106, CA, USA; e Department of Molecular, Cellular and Developmental Biology, University of California Santa Barbara, Santa Barbara, 93106, CA, USA; f Department of Molecular, Cell, and Developmental Biology, University of California Santa Cruz, Santa Cruz, 95064, CA, USA; g Department of Computational Media, University of California Santa Cruz, Santa Cruz, 95064, CA, USA

**Keywords:** Internet of things, Cloud biology, Biotechnology, Stem cells, Brain organoid, Neural development

## Abstract

The analysis of tissue cultures requires a sophisticated integration and coordination of multiple technologies for monitoring and measuring. We have developed an automated research platform enabling independent devices to achieve collaborative objectives for feedback-driven cell culture studies. Our approach enables continuous, communicative, non-invasive interactions within an Internet of Things (IoT) architecture among various sensing and actuation devices, achieving precisely timed control of *in vitro* biological experiments. The framework integrates microfluidics, electrophysiology, and imaging devices to maintain cerebral cortex organoids while measuring their neuronal activity. The organoids are cultured in custom, 3D-printed chambers affixed to commercial microelectrode arrays. Periodic feeding is achieved using programmable microfluidic pumps. We developed a computer vision fluid volume estimator used as feedback to rectify deviations in microfluidic perfusion during media feeding/aspiration cycles. We validated the system with a set of 7-day studies of mouse cerebral cortex organoids, comparing manual and automated protocols. It was shown that the automated protocols maintained robust neural activity throughout the experiment while enabling hourly electrophysiology recordings during the experiments. The median firing rates of neural units increased for each sample, and dynamic patterns of organoid firing rates were revealed by high-frequency recordings. Surprisingly, feeding did not affect the firing rate. Furthermore, media exchange during a recording did not show acute effects on firing rate, enabling the use of this automated platform for reagent screening studies.

## 1. Introduction

Recently, advances in biological research have been greatly influenced by the development of organoids, a specialized form of 3D cell culture. Created from pluripotent stem cells, organoids are effective *in vitro* models in replicating the structure and progression of organ development, providing an exceptional tool for studying the complexities of biology [1,2]. Among these, cerebral cortex organoids (hereafter “organoid”) have become particularly instrumental in providing valuable insights into brain formation [3–5], function [6,7], and pathology [8,9]. Electrophysiological measurements have been used to characterize neuronal function in organoids [10–14], revealing neuronal network properties consistent with early fetal brain development. Organoid models are constantly improving through new protocols for different areas of the brain [4,15–18], the fusion of organoids from different brain areas (assembloids) [7,19,20] as well as connectivity of multiple organoids through axonal bundles (connectoids) [21,22], and integration of missing components such as microglia [23–25] and vascularization [26–28].

Despite their potential, organoid experiments present significant challenges. Brain organoids require a rigorous, months-long developmental process, demanding substantial resources and meticulous care to yield valuable data on aspects of biology such as neural unit electrophysiology [29], cytoarchitecture [30], and transcriptional regulation [9]. The primary methods for generating and phenotyping organoids depend on media manipulations, imaging, and electrophysiological measurements [31,32], which are all labor- and skill-intensive, limiting the power and throughput of experiments [33]. Cell culture feeding and data collection occur at intervals realistic for researchers. Furthermore, during manual feeding and data collection, the cell cultures are removed from the incubator, which provides a controlled gas, temperature, and humidity environment [34]. Ideally, feeding should be aligned with the cells’ metabolic cycles, and data should be collected at intervals on par with the biological phenomenon. The disturbance incurred by leaving the incubator environment is shown to increase metabolic stress and batch-to-batch variability, potentially impacting the quality of the experiment [35], as well as increasing contamination risk.

Laboratory robotics, most often liquid handling devices [36], offer increased precision and throughput but are primarily designed for pharmaceutical screens, limiting their adoption in research labs due to high costs, large footprints, and inflexible workflows [37]. Moreover, many of these systems lack the ability to seamlessly integrate new technologies as they emerge. Conversely, academic research labs are benefiting from advancements in commercial and custom-made technologies, facilitated by in-house fabrication methods like 3D printing [38–40], which are enhancing their capacity to manipulate and measure biological systems. However, without an easy-to-integrate, device-agnostic robotic platform, researchers are constrained to manual operations, restricting the power and scope of their experiments. By outfitting devices to carry out automated jobs and relay data through communication networks, they acquire around-the-clock functionality and increased fidelity [41]. Flexibility in size (number of devices per integrated system) allows researchers to optimize for the experimental design and budget. Implementing programmable feedback loops derives precision and self-optimization by dynamically adjusting to real-time data [42–44], offering a practical alternative to complex mathematical modeling for experiment control. This approach would enable more integrated, flexible automation in research settings, broadening the scope and efficiency of experiments.

Automating multiple devices to report data presents a challenge for device management and communication, necessitating flexible and efficient infrastructure. Addressing this need for an interconnected ecosystem of devices, services, and technologies is possible through designing networks using standards defined by the Internet of Things (IoT). IoT has already impacted agriculture [45], city infrastructure [46,47], security [48], and healthcare [49–53]. Applications in biology are emerging in education [54–58] as well as research [59–61], but most systems lack integration and collaboration between multiple instruments. Recent work [49] illustrates connected patient monitoring systems, while load management strategies in IoT [46] highlight parallels to biological feedback regulation. These developments inform our platform’s approach to synchronizing automated biological instrumentation.

The main objectives of this study were to develop an integrated, IoT-enabled platform capable of automated feedback-driven control of brain organoid culture, and demonstrate its efficacy by comparing the outcomes of automated and manual protocols using electrophysiological readouts. Previously, each researcher built a custom device and code from scratch with unique assumptions for communication and behavior. Each device operated in solitude, lacking integration and feedback with other devices. Here, we establish a platform that addresses these challenges, combining electrophysiology, microscopy, microfluidics, and feedback control, automated and integrated through IoT technology for touch-free, in-incubator tissue research. Our system addresses these issues through modular, low-footprint automation, real-time IoT integration, and improved in-incubator experimental control.

## 2. Results

### 2.1. An integrated microfluidic, electrophysiology, and imaging organoid research platform

**Fig. 1.**
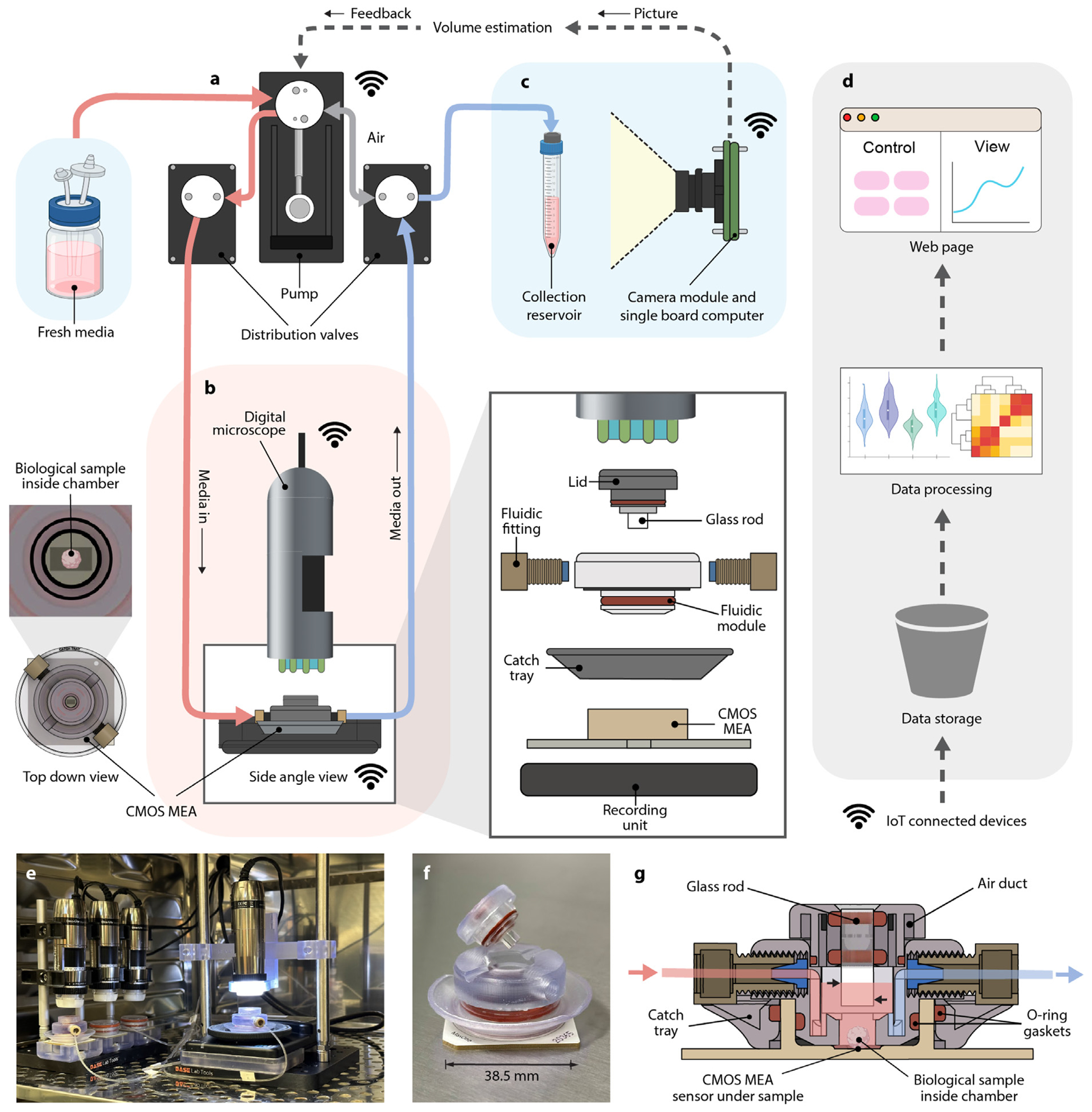
Schematic diagram of the integrated feedback platform. (a) A syringe pump and valve system dispenses fresh media and aspirates conditioned media at user-defined intervals. The blue background represents 4 °C refrigeration. (b) Microscopy and high-density microelectrode array (HD-MEA) electrophysiology record morphology and functional dynamics of the biological sample. The red background represents 37 °C incubation. Exploded view: the microfluidic culture chamber for media exchange couples with the HD-MEA. (c) A camera captures images of the aspirated conditioned media drawn from each culture and relays them through cloud-based data processing for volume estimation feedback to the syringe pump system. (d) Devices communicate over Message Queuing Telemetry Transport (MQTT) protocol and automatically upload data to the cloud, where it is stored, processed, and presented on a web page. (e) The experimental setup in the incubator shows two microfluidic culture chambers and two conventional membrane lids. (f–g) 3D printed microfluidic culture chamber and cross-section diagram. The media level, noted by the upper black arrow (559 μL) and lower black arrow (354 μL) on the glass rod, is the ideal operating range that keeps the rod immersed in media. The biological sample is adhered to the HD-MEA sensor at the bottom.

We have developed an integrated platform (Fig. 1) that automates organoid culture and data collection in individual microenvironments. While microfluidics (Fig. 1a) controls the media environment, digital microscopy captures the morphogenic features. The neural activity is recorded by local field potential measurements using complementary-metal-oxide semiconductor (CMOS) high-density microelectrode arrays (HD-MEA) [62] (Fig. 1b). The Internet of Things (IoT) cloud network brokers the communication between all devices and facilitates data storage, processing, and presentation services including an interactive webpage (Fig. 1d). Through touch-free automation, samples remain undisrupted in the incubator, increasing the consistency of images and allowing for higher frequencies of feeding and recording.

At user-defined intervals, conditioned media is aspirated by a syringe pump through a system of distribution valves (Fig. 1a), stored in a collection reservoir (without passing through the syringe pump vial) (Fig. 1c), and replaced by an equivalent volume of fresh media. Both types of media are perfused through flexible fluorinated ethylene propylene (FEP) tubing at 110 mm/s, which leads to low shear forces [63] (see Materials and methods, Microfluidic cell culture). This equates to a flow rate of 44.1 μL/s.

The digital microscope (Fig. 1e) is attached using 3D-printed parts on aluminum posts. The 3D printed culture chambers integrate the microfluidics and HD-MEAs. A liquid-impermeable O-ring gasket ensures media retention inside the chamber. The well lid includes a polished glass rod submerged in the media, improving image quality and removing the effects of condensation. Alignment grooves in the glass rod lid prevent rotation and incorrect fitting. The lid exchanges gas with the incubator conditions through ventilating air ducts (Fig. 1g), similar to a cell culture well plate. The removable and re-attachable lid reduces manufacturing complexity and enables future use of other lids with applications beyond imaging.

Fig. 1c shows the cross-section of the culture chamber attached to the HD-MEA. The media flows in (red) and out (blue). The sinuous media path and well geometry ensure minimum disturbance to the biological sample [63]. Fresh media is delivered on top of the volume present in the chamber, similar to partial media changes found in manual feeding protocols [64,65]. The ideal operating range is between 350 to 700 μL (see Supplementary Materials and Methods, Figure 1 and Table 1 for numerical volume limits). In the case of over-aspiration, media drops to a minimum of 170 μL before aspirating air from the chamber’s headspace. The 3D-printed catch tray guards against overflow, collecting up to 1.5 ml (200% of the chamber’s capacity) to protect the recording equipment from liquid damage.

### 2.2. Computer vision for microfluidic flow feedback

**Fig. 2.**
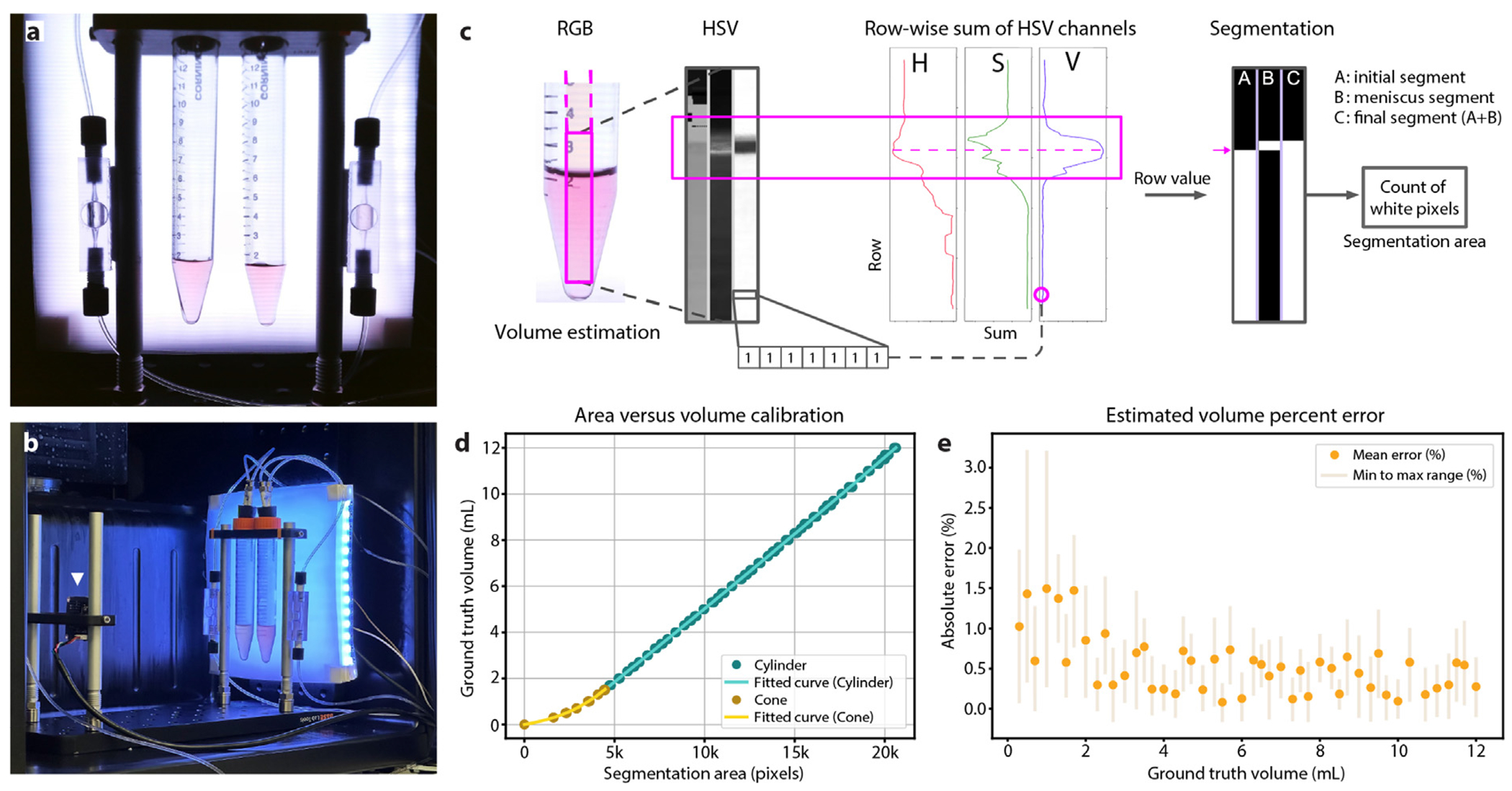
Computer vision for volume estimation. (a) Example of a raw image captured by the camera module. (b) In-refrigerator volume estimation setup in Fig. 1c. The CMOS camera module (the white triangle) images the conical tubes with a diffused LED backlight for even illumination. (c) Fluid segmentation: a rectangular pixel patch down the center of the conical tube; Row-wise summations of the HSV channels are used to detect the location of the meniscus. These summations emphasize vertical gradients capable of detecting the air–liquid boundary even with image noise or uneven illumination. The resulting meniscus estimate is combined with an initial fluid segmentation pass to form the final volume segmentation mask. (d) Calibration graph with a fitted relationship of segmented pixel count to ground truth volume. (e) The absolute error percentage: orange dots represent the average error at selected volumes. The shaded bar represents the minimum to maximum error range.

We developed a computer vision volume estimation system to monitor the accumulation of aspirated media and identify anomalies during culture feeding events. A critical technical challenge arose from media crystallization and debris accumulation, which could obstruct microfluidic lines during aspiration. We resolved this by implementing a computer vision-based feedback loop that flagged incomplete aspiration cycles, triggering corrective actions until the target volume was achieved or user assistance was requested. These safeguards enabled autonomous error recovery and prevented feed cycle failure throughout the study. Fig. 2a–b provides a detailed view of the setup inside a refrigerator, which includes three main components: a collection reservoir support system, an LED panel, and a camera module (see Materials and methods, 3D-printed components). The camera module remains on standby for image capture requests made by other IoT devices or users. Upon request, computer vision techniques are employed to estimate the media volume within the reservoirs accurately. This circuit converts camera-captured images into quantitative volume measurements in real time, enabling closed-loop feedback to the pump during culture maintenance.

Fig. 2c shows the computer vision process (see Materials and methods, Computer vision for fluid volume estimation) for segmenting area related to the media in the reservoir. A calibration was required to establish the relationship between the segmented area in pixels and volume in milliliters. We captured 184 images of the collection reservoirs containing volumes of media ranging from 0 to 12 mL (several pictures for each volume), with each volume confirmed by a scale, accurate to 1 μL. This calibration process yielded a robust polynomial mapping between segmented pixel area and true volume. We trained two regression models to account for the conical and cylindrical regions of the reservoir, achieving high fidelity across the full 0–12 mL range. For each specific volume in Fig. 2d, multiple points overlap and are all accounted for to calculate the polynomial regression lines. To accommodate the reservoir’s conical section (volumes <1.5 mL) and cylindrical section (volumes >1.5 mL), two distinct regressions were applied, ensuring a high degree of precision for each geometrical shape.

A Leave-One-Out cross-validation (LOO) [66] approach was employed to quantify the model’s error. This method tests the model’s accuracy and generalizability in an unbiased manner, ensuring that the calibration results in a model that performs reliably across different samples. The effectiveness of the model is assessed quantitatively with the following metrics: an average Mean Absolute Error (MAE) of 0.56% (equivalent to 27 μL), an average standard deviation of errors at 0.53% (22 μL), and an average Root Mean Square Error (RMSE) of 0.77% (35 μL). The polynomial models exhibit R-squared values of >0.99, denoting an optimal fit of pixel area to liquid volume. Fig. 2e shows the average absolute error percentage at a specific volume, with the bar indicating the error range from minimum to maximum.

### 2.3. IoT infrastructure creates an ecosystem of devices and cloud-based services

**Fig. 3.**
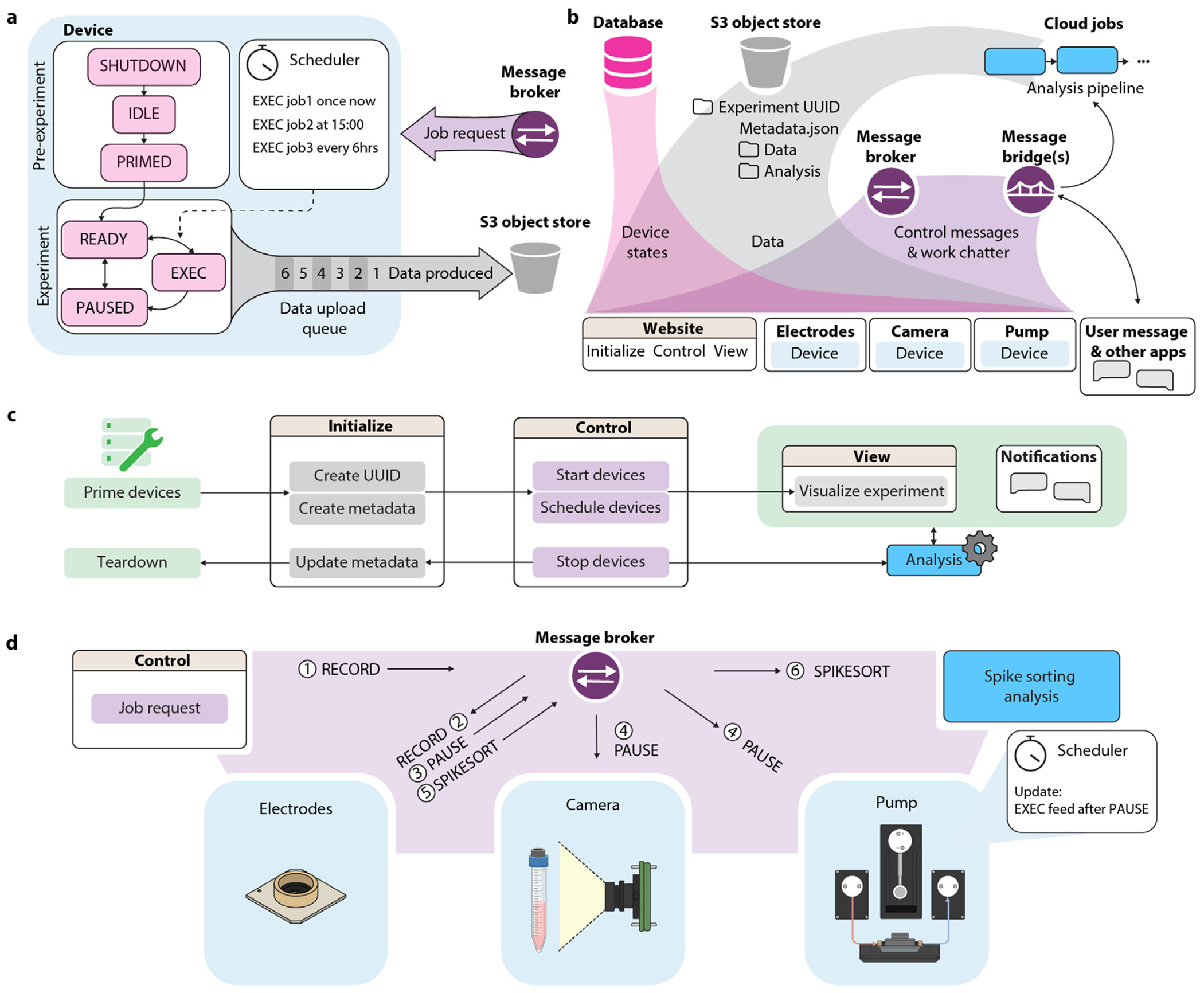
Cloud-based device interactions. (a) The *device-class* is a generalized state machine framework of all IoT devices. The *device* participates in experiments by taking in job requests (from experimenters or other devices), scheduling and executing the jobs, and producing data files that are queued and uploaded to cloud storage. (b) IoT infrastructure. Device states (pink) are saved in a database and displayed on the website user interface. Device-generated data (gray) is saved and organized in cloud storage, where it can be accessed by user interface or analysis cloud jobs. Devices send communications (purple) through a message broker and use message bridges to translate messages to analysis pipelines or text messaging applications. (c) User workflow. Devices are physically primed in accordance with experimental procedures such as sterilization. On the ‘Initialize’ webpage, an experiment is created with a unique ID (UUID) and descriptive notes (metadata). On the ‘Control’ webpage, devices are called to start working on the experiment and are given a job schedule. The ‘View’ webpage and notifications allow the user to monitor the ongoing experiment. (d) Example of inter-device communication: (1) A RECORD job request is made from the ‘Control’ panel. (2) The message broker delivers the record request to the electrophysiology recording unit. (3) The electrophysiology unit pauses all other devices to ensure a quality recording. (4) All devices receive a pause request. The pump reschedules a feed until after the pause. (5) Upon finishing the recording, the electrophysiology unit delivers a spike sorting request to commence data analysis.

We built a cloud-based IoT ecosystem that enables communication between users, devices, and services to implement actions, record data, and streamline upload, storage, and analysis. All devices (here: pumps, microscopes, and microelectrode arrays) run software using the *device-class* Python framework (Fig. 3a and Supplementary Materials and Methods). Devices operate collectively with shared core software and complementary behaviors: they can request jobs from each other, yield during sensitive operations, and ensure collaborative functions and smooth operation (Fig. 3d). One challenge during multi-device coordination was managing asynchronous job execution across devices (e.g., electrophysiology, fluidics, imaging). We addressed this by implementing a device-level state queue that logs jobs and defers them opportunistically, ensuring time-sensitive tasks (e.g., recordings) were uninterrupted while preserving non-conflicting tasks (e.g., uploading). This prevented job loss and allowed error-tolerant, coordinated automation across modalities. Devices update their *shadow* in the database whenever their state information changes (i.e, assigned experiment, schedule, current job and estimated completion time, and other dynamic variables) to eliminate the need for device polling. Messages (i.e., job requests) between devices and services are sent through a centralized MQTT broker via the publish/subscribe protocol. This decoupled architecture allows for independent and extensible deployment of components. Data generated by devices is immediately uploaded to an S3 object storage in a predefined structure using an experiment Universally Unique IDentifier (UUID) as the top-level key. Uploading large electrophysiology datasets presented bandwidth-related challenges due to inconsistent internet connectivity. We developed a file queue system with automatic retry and checksum validation to ensure upload fidelity and prevent data corruption or loss during transfer. A ‘metadata.json’ file stores experiment details, sample information, notes, and an index of the produced data. Raw data is stored separately from analyzed data under different sub-keys. Cloud jobs, which operate as shared services, process raw data from S3 and write results back to S3, reporting status via MQTT messages. To utilize the IoT ecosystem, users initiate experiments, control devices, and visualize data through a website (see Materials and methods, Website and screenshots in Supplemental Figure 2), with the typical user workflow in Fig. 3c.

### 2.4. Automated study of cerebral cortex organoids

The integrated research platform was used to study the effects of automation on the neuronal activity of pluripotent stem-cell-derived mouse cerebral cortex organoids. Embryonic stem cells were aggregated, patterned, and expanded to generate organoids using a previously defined differentiation protocol [57,67]. Day 32 post-aggregation, organoids were plated two-per-chip directly onto HD-MEAs. Two were plated to maximize use of the HD-MEA surface (3.85 × 2.10 mm^2^). For the 7-day study, 8 chips across two batches were split into groups that were fed and recorded with standard manual procedures (Controls, N = 4), manual feeding and automated recording (AR, N = 1), automatic feeding and manual recording (AF, N = 1), or automatic feeding and automatic recording (AFAR, N = 2). Manual feeding for the Control and AR groups involved aliquoting and pre-warming fresh media within a biosafety cabinet, transferring the culture from the incubator to the cabinet, aspirating conditioned media by pipette, replenishing with fresh media, and returning the sample to the incubator. Automated feeding for the AF and AFAR groups involved microfluidic aspirating of conditioned media and microfluidic dispensing of fresh media while the sample remained undisturbed in the incubator. All chips were imaged in the incubator every hour, each using a dedicated upright digital microscope (DinoLite).

**Fig. 4.**
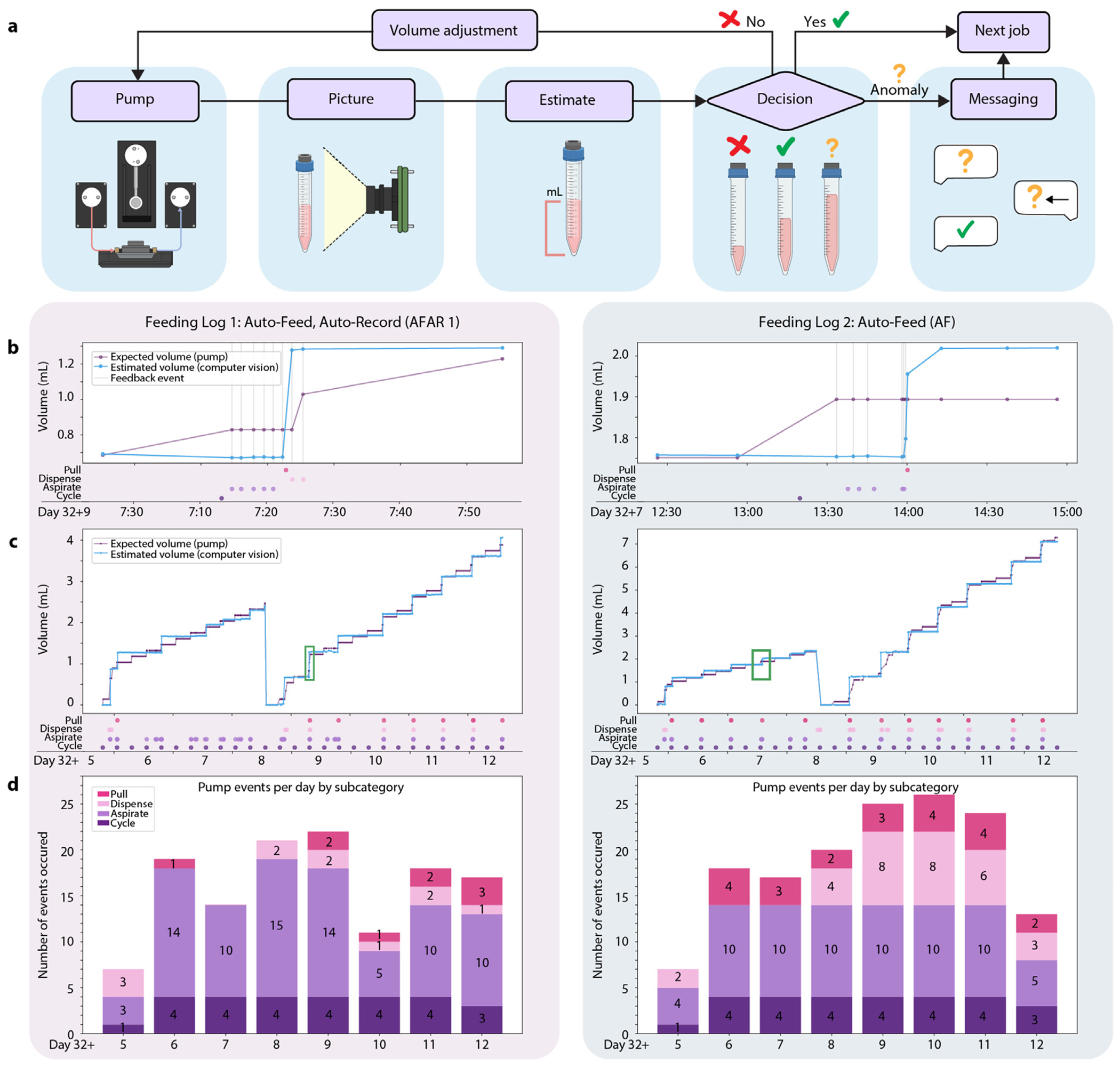
Volume feedback. (a) Volume estimation feedback loop. After the pump completes a microfluidic action, it requests a picture of the media collection reservoir from the camera module. The picture is passed to the cloud-based computer vision program to estimate the current volume. The result is compared with the expected volume, and a decision is made: within tolerance (green checkmark), a microfluidic volume adjustment action is needed (red “x”), or an anomaly is detected (yellow question mark). Once the estimated volume is within tolerance (green check mark), the feedback cycle ends and proceeds to the next job. If this cannot be achieved or an anomaly is detected, such as out-of-range volumes, an alert is sent to the user messaging service to request assistance. (b–d) On these graphs, the “Day” *x*-axis summarizes the timeline: organoids were plated on the HD-MEA on Day 32, automation started 5 days after plating and continued to day 12. Above this axis, dots mark the occurrence of microfluidic events. (b) Graphs of the Expected Volume and Estimated Volume for the automated Auto-Feed, Auto-Record replicate 1 (AFAR 1) (left) and Auto-Feed (AF) (right) during a period of feedback events. Event types are marked with dots below the graph. (c) The complete view of Expected and Estimated volume traces over the 7-day study. (d) Stacked histogram pump events per day organized by type (Pull, Dispense, Aspirate, and Cycle). This showcases the variability of feedback events required to retain adherence to the feeding regime.

Automated microfluidic feeds were used to increase the consistency and frequency of cell culture media replacement. We removed conditioned cell supernatant from the well and dispensed the equivalent volume of fresh media for each feed cycle. The controls had 1.0 mL media replacement every 48 h, consistent with standard protocols. AF and AFAR were placed on a protocol in which 143 μL media were replaced every 6 h, matching the total media volume turnover across groups for the 7-day study. The schedule of automated media feeds was defined at the experiment’s launch and initiated by a timed feeding job command sent to the microfluidic pump. The fidelity of feeding was controlled through a computer vision volumetric feedback loop on the aspirated conditioned media (Figs. 2, 4a). To assess operational efficiency, we quantified feedback performance across microfluidic feeds. The system was designed to maintain a strict schedule of four feeds per day (28 total replenishment cycles over the 7-day study). For AFAR 1, 61% of the scheduled replenishment cycles (17 of 28) triggered feedback corrections, comprising 81 aspiration, 11 dispense, and 9 pull actions. Feedback events per trigger ranged from 1 to 10 with a mean of 6. For AF, 54% of replenishment cycles (15 of 28) required feedback, with 69 aspirations, 31 dispenses, and 22 pulls, and a slightly higher mean of 8 feedback actions per trigger (range 2–11).

Conditioned media has a high protein content, contains cellular debris, and is susceptible to forming salt crystals [68,69]. In microfluidic systems, this leads to clogs, error accumulation, and failure modes [70]. To overcome this, a volume estimation feedback loop was initiated each time the pump performed a job. At the time the medium was perfused to/from a specific well, the pump sent a job request to the camera module responsible for imaging the well’s collection reservoir. The image was captured, uploaded to the cloud, its volume estimated by the computer vision Estimator, and returned to the pump for feedback interpretation. The pump compared this measured volume with its expected target and responded accordingly: initiating further aspiration or dispensing jobs if a discrepancy exceeded the tolerance range, or logging success if the measurement matched. This closed-loop interaction occurred autonomously without user intervention, with exception handling only triggered if repeated corrections failed. Within tolerance, the action was declared a success (marked as a green check mark in Fig. 4a), and no further action was taken. Outside of tolerance, the pump scheduled itself a new job proportional to the volume discrepancy and in relation to the number of previous feedback attempts (see Materials and methods, Feedback interpreter).

The system was designed to resolve errors in media exchange using feedback. If feedback actions exceed set thresholds, which were perfusion of >2 mL or >20 retry attempts, the system sends an alert via Slack and halts operations. This was designed to prevent overflow or underflow events and ensure media volumes remain within tolerance. In both 7-day experiments, no interventions were required.

The automated feeding and feedback results for AF and AFAR 1 are visually represented in Fig. 4b–d. Fig. 4c shows the traces of expected volume and computer vision estimated volume for AFAR 1 (left) and AF (right) for the 7-day study (Days 5 to 12 post-plating). There was a collection reservoir change on Day 8 in which the 15 mL conical was replaced with a fresh tube. In both samples, the drop in estimated and expectation reflects the collection reservoir exchange. For AFAR 1 (Fig. 4b, left), a zoomed-in view of the feedback loop following the scheduled feeding cycle at 7:12 on Day 9 highlights feedback actions taken to remedy a volumetric discrepancy. In this instance, the volume estimation was less than expected after the feed cycle. Five consecutive aspiration jobs were carried out, and the estimated volume still remained under expectation. At the 6th iteration of feedback, a pull job was sent to the pumps, which raised the collection volume above the expected volume. In the 7th and 8th iterations of feedback, two dispense jobs were engaged to supplement the well for the over-aspiration. In a similar case, for AF (Fig. 4b, right), a total of 6 iterations of feedback were engaged to bring the estimated volume into tolerance with the expected volume; however, in this example, no dispense jobs were required. Fig. 4d shows histograms of the sum of pump events per day by subcategory. Each feeding cycle (four per day) was scheduled, and all other events occurred through feedback.

### 2.5. High-frequency HD-MEA recordings and automated feeding do not disrupt neuronal activity

**Fig. 5.**
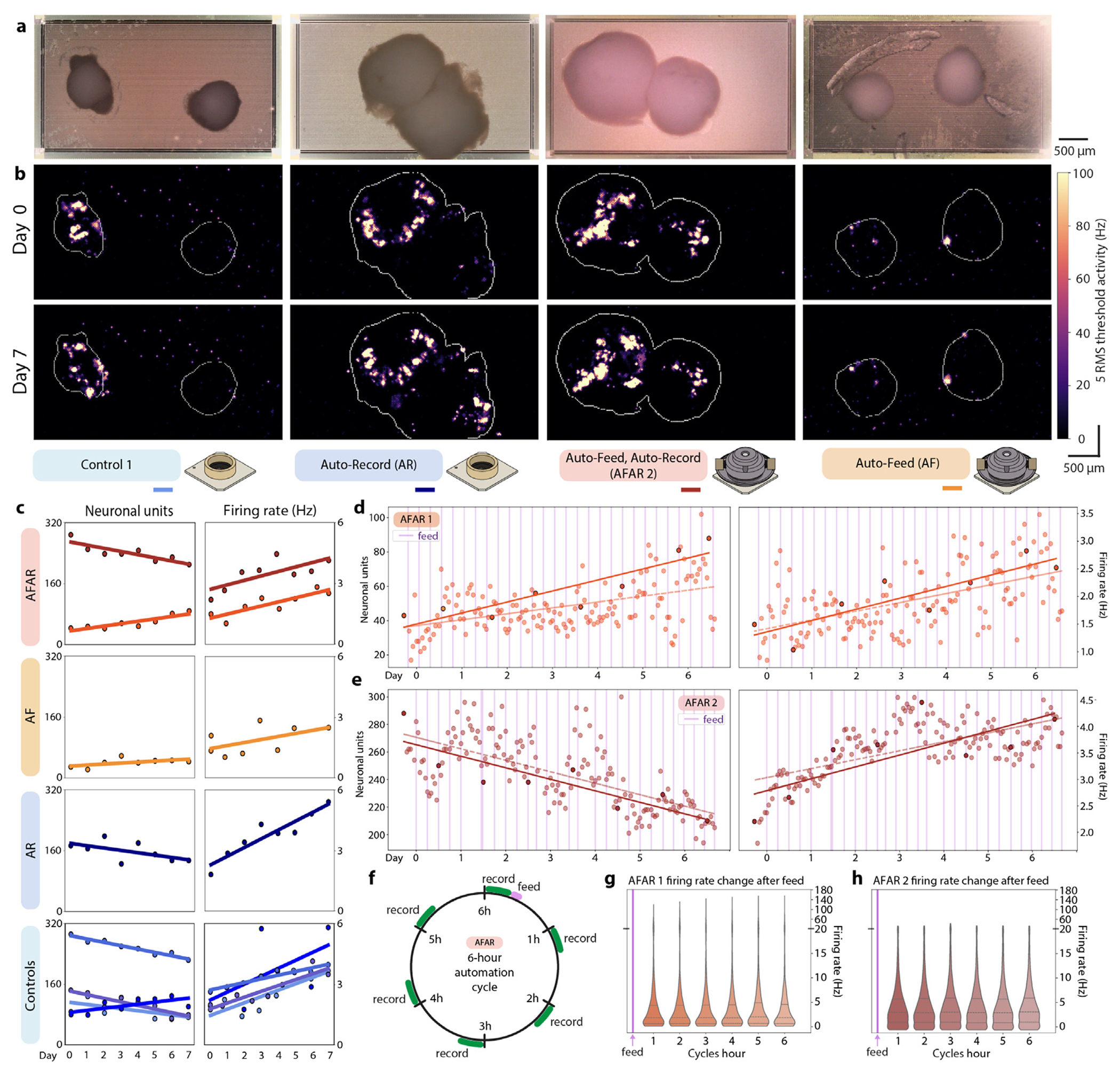
Electrophysiology analysis of the 7-day cerebral cortex organoid study. (a) Digital microscope images of example organoid conditions. (b) Boundaries of each organoid were outlined using image segmentation and overlaid with activity scans from the initial recording “Day 0” (top) and last recording “Day 7” (bottom). Experimental conditions are labeled underneath, with a color legend. (c) Total detected neural units (left column) and median firing rates per unit (right column) in daily 10-min recordings, grouped by experimental condition. (d–e) Detected neural units (left) and median firing rates (right) in hourly resolution for Auto-Feed, Auto-Record replicate 1 (AFAR 1) (d) and Auto-Feed, Auto-Record replicate 2 (AFAR 2) (e) using their automated 10-min recordings. Feeding events are noted as vertical violet lines. (f) The two AFAR samples had a 6-h automation cycle that included one 143 μL feed (violet) and six 10-min recordings (green). (g–h) Violin graph of all units’ firing rates per recording for AFAR 1 (g) and AFAR 2 (h) organized into bins of the 6-h automation cycle following each feeding event. The 6-h feeding regimen did not induce cyclical changes for either sample.

To evaluate organoid neuronal activity, extracellular field potentials were measured using 26,400 electrode HD-MEAs, which can record up to 1020 electrodes simultaneously. We conducted daily activity scans to monitor neural activity. Activity heat maps derived from the first and final activity scans for each sample are presented in Fig. 5b, with an outline of the organoid edge based on alignment of corresponding microscopy images. To optimize electrode coverage, we generated specific configuration files for electrode selection based on the regions with the highest activity, which remained constant for seven of the eight chips. In one case (AF, Day 32+6), we updated the configuration due to the emergence of a new high-signal area on the second day of recording. Stable configuration maps allowed for automated electrode recordings over days, optimizing long-term analysis.

Manual recordings involved an experimenter placing each HD-MEA on the recording unit and initiating 10-min recordings via software. In contrast, the hourly recordings (AFAR 1-2 and AR) featured the HD-MEA remaining on the recording unit while automated software handled the entire process, from power management to data uploading. AFAR 1-2 and AR each amassed over 158 recordings, totaling over 26.3 h (525 GB) of electrophysiology data per sample. Conversely, all manually recorded samples (Controls 1-4 and AF) accumulated 7 recordings each, amounting to 1.2 h (23 GB) of electrophysiology data. The studies generated over 2 TB of data from over 100 h of mouse organoid recordings.

From these data, we analyzed the effects of our automated microfluidic, imaging, and recording system on the neuronal activity of the brain organoids housed therein. Imaging of the chips from above (Fig. 5a) allowed us to align the body of the organoid with neural activity (Fig. 5b). In some instances, such as in Control 1, neurite outgrowths were evident in the images and activity scans.

Initial activity scans were used to distribute samples into experimental and control conditions. We sought an even distribution with respect to baseline activity to reduce biases from initial conditions. Groups were assigned based on median unit counts and neural activity. Both Controls and Automated samples included organoids with fewer than 100 units and organoids with greater than 100 units. Samples with fewer than 25 units were excluded to ensure reliable electrophysiology analysis. Neural units identified from initial recordings by spike sorting with Kilosort2 [71] were: Control 1: 87 units, Control 2: 292 units, Control 3: 144 units, Control 4: 80 units, AR: 173 units, AFAR 1: 43, AFAR 2: 250, AF: 29. Three chips were omitted from the study for having a unit count less than 25. Throughout the 7-day study, all samples with unit counts 29 to 80 (Control 4, AFAR 1, and AF) increased their detected unit counts during the 7-day study, while all samples with unit counts 87 and above (Control 1-3, AR, and AFAR 2) decreased their detected unit counts (Fig. 5c).

The average firing rate per neural unit increased for every sample, irrespective of feeding or recording schedules (Fig. 5c). All samples started with an average firing rate of 1.95 Hz (σ=0.48), increased by 0.24 Hz per day (σ=0.09), and concluded with an average of 3.98 Hz (σ=1.22). The automated conditions (AR, AFAR, and AF) presented no divergence from the controls on neural unit count, firing rate, or morphology resulting from increased frequency of feeding and/or increased frequency of recording.

### 2.6. High-frequency HD-MEA recordings reveal dynamic neuronal activity states in organoids

The hourly recorded conditions (AFAR 1-2 and AR) revealed transient states, not apparent with single daily recordings (Fig. 5c–e). Linear regression trendlines comparing the hourly and daily recordings for a single sample are congruent, however daily recordings do not capture the prominent oscillatory dynamics of neuronal unit count and median firing rate captured by the hourly recordings. Median firing rates were observed to fluctuate as much as 3-fold over the course of a day and are not well-characterized by linear regression fit (AFAR 1 *R*^2^ = 0.31, AFAR 2 *R*^2^ = 0.42, AR *R*^2^ = 0.69).

We inspected the effect of feeding on these dynamics. The AFAR samples had a six-hour automation cycle (Fig. 5f) that included one 143 μL feed and six 10-min recordings. We examined effect by aligning recordings to a six-hour ‘time since feed’ cycle. Fig. 5g and h present the composite graphs of aggregated neuronal firing rates of AFAR 1 and AFAR 2, comprising 26 feeding cycles with all recordings binned with respect to their time since feeding. The superimposed feeding cycles did not show a trend in units (not shown) nor firing rate in relation to feeding cycles. The variance presented in Fig. 5d and e do not align with the six-hour feeding cycle.

### 2.7. Effects of feeding during recording

**Fig. 6.**
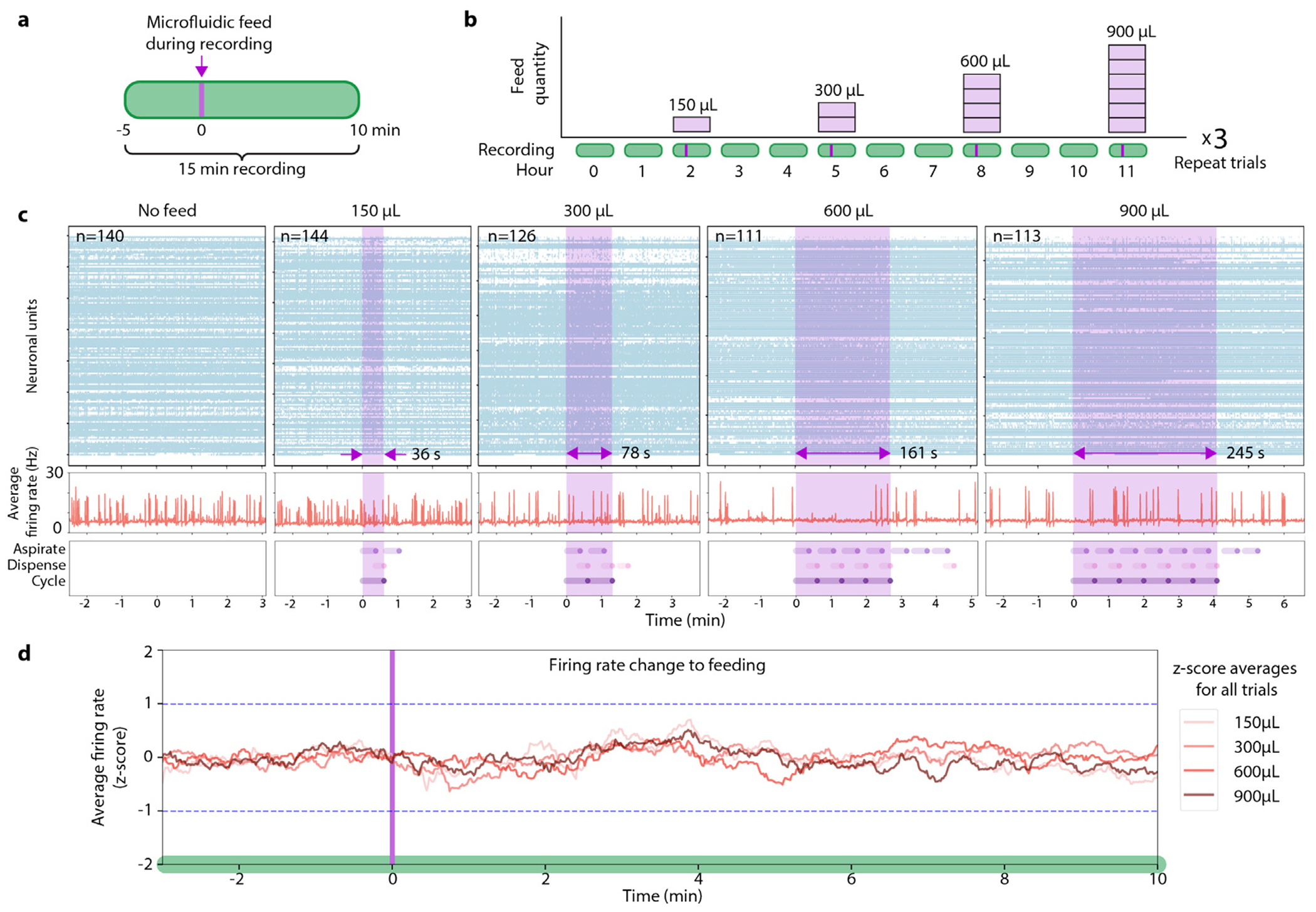
Effect of feeding during recording. (a) 15-min recordings were collected hourly from a cerebral cortex organoid, with automated microfluidic feeding beginning at minute 5. (b) Feedings occurred every third hour of ascending fixed-volume cycles of 150 μL, 300 μL, 600 μL, and 900 μL, then repeated for three trials (36 h). (c) Graphs of select recordings showcasing each of the five conditions. Top: spike raster of neuronal units (y axis) firing over time (x axis). Middle: average firing rate per neuronal unit computed by dividing the total spikes in a 100 ms bin by number of neuronal units. Bottom: microfluidic feeding actions performed by the pump. Each feeding “cycle” is composed of one “aspirate” action followed by one “dispense” action. No “pull” actions occurred in the graphs. Pink shading in the Top and Middle graphs represents the summed feeding duration, while the Bottom graph breaks down the specific pump actions performed. Additional actions triggered by feedback are outside the pink feeding window. (d) Firing rate dynamics in response to feeding events across ascending cycle conditions. Neural activity was analyzed using 90-s sliding windows (in 1-s steps) and normalized in two stages: first using contrast normalization (‖x‖=(a−m)/(a+m), where ‖x‖ is the norm, *a* is the firing rate of a neural unit in each window and *m* is the mean firing rate across all windows for that unit within its recording) to account for individual unit firing rate differences, followed by z-score normalization at each time window against the no-feed control recordings to account for natural baseline firing variability. Z-scores were averaged across units within each feed volume condition (150 μL to 900 μL) to evaluate the influence of progressively larger feed volumes.

We further investigated the effect of feeding with a follow-up experiment that included microfluidic feeds during electrophysiology recordings to capture the immediate response on neuronal activity to a media injection. The AR sample from the 7-day study (Fig. 5) was equipped with a microfluidic culture chamber and set on a new feeding and recording schedule. Automated 15-min recordings were performed every hour for 36 h. Feedings occurred every third hour that began at minute 5 of the ongoing recording (Fig. 6a). Each feeding cycle was defined as an aspiration and dispense of 150 μL. Automated feeds increased in their number of cycles each third hour for four experimental conditions (1 cycle = 150 μL, 2 cycles = 300 μL, 4 cycles = 600 μL, and 6 cycles = 900 μL). Each of these conditions were performed three times (Fig. 6b).

Fig. 6c presents neural unit raster plots and the average neuron firing rate for representative recordings of each condition. Raster plots of neural unit firing over time show no change in unit activity during microfluidic manipulation (light purple overlay). The average firing rate did not show unusual variability during or after the feeding window and did not correlate to pump actions (Dispense, Aspirate, or Cycle). To further inspect this computationally, we performed normalization and z-score analysis (detailed description in Materials and methods, Normalization and z-score calculation for effect of feeding during recording). Within each recording, a 90-s sliding window was applied to the spike raster with 1-s steps. The firing rate of a single unit at any particular window was normalized to the same unit’s average firing rate across all windows within a recording. This scales changes in activity for any neural unit to be comparable irrespective of average firing rate. For all non-feeding recordings (N = 28), we calculated the firing rate mean and standard deviation (STD) of all normalized units in each window. The results of the experimental conditions (Fig. 6d) (N = 3 per condition) were calculated with z-scores generated for each neural unit in each window to relate how much firing rate changed with respect to baseline variability. The STD of ±1 in relation to the non-feed activity are marked (dashed blue line). In the 10 min following the onset of microfluidic feeding, the largest increase in firing rate was +0.7 STD above the mean at 3.9-min (150 μL condition), largest decrease was 0.6 STD below the mean at 0.8-min (300 μL condition). Z-scores for all conditions (150 μL, 300 μL, 600 μL, and 900 μL) remained within ±0.7 STD showing no significant change in activity during microfluidic manipulations.

Feed cycles of different total volume and time length did not elicit immediate changes in firing rates during or minutes after feeding. Combined with our findings in Fig. 5 showing no changes in firing rate distributions for six hours following feeding cycles, these results suggest that neural activity remains stable despite media exchange and the associated fluid movement.

## 3. Discussion

This automated platform advances organoid research methodology, enabling continuous monitoring and manipulation of brain organoids while maintaining optimal cell culture conditions through non-disruptive, automated protocols. It addresses issues of manual handling, variability, and limited temporal resolution. Automated conditions collected >150 recordings per chip with no failed feeds or user interventions. Manual conditions averaged 7 recordings with higher variability. Feedback events corrected minor flow errors autonomously, reducing user time and increasing experimental reproducibility. By integrating microfluidics, electrophysiology, and imaging through an IoT framework, we have created a system that supports experimental reproducibility and reveals temporal dynamics previously difficult to capture in manual protocols. Running on a distributed IoT network offers dual benefits: a local MQTT broker ensures reliable performance even during internet outages while Cloud integration enables global collaboration across distant labs for shared or complementary research. This setup enhances the continuity of individual experiments and the integration of worldwide scientific efforts. The reduction of human intervention enabled by the microfluidic feeding system reduces the risk of contamination, variations in time outside the controlled temperature and CO2 incubator environment during feeding and imaging, and other human-introduced variances. This degree of control is particularly valuable in long-term organoid experiments toward reducing batch effects.

Automated feedback mechanisms provide essential experimental control by maintaining conditions within defined target ranges without manual supervision. In this study, we implemented a computer vision feedback loop to maintain consistent media volume within the organoid growth chamber, ensuring stable environmental conditions across time. During our 7-day studies, the system achieved this feedback autonomously and did not require manual intervention to rectify anomalies. We seek to expand implementations of feedback in future work by using real-time electrophysiological signals, morphological growth patterns from imaging, and optical detection of media analytes (e.g., pH or metabolites) as input triggers. These could control actions such as initiating electrical stimulation, altering feeding frequency, or injecting compounds in response to specific biological states. To improve robustness, future implementations could incorporate adaptive error handling strategies such as timeout thresholds, confidence-weighted sensor fusion, and automated fallback routines. These would allow the system to autonomously switch modes, reassign jobs, or alert users when predefined limits are exceeded during feedback operations. Devices can use the flexibility of MQTT messaging to allow for the creation of additional feedback loops to control the experiment. The computer vision infrastructure could also support absorbance or colorimetric assays, expanding the system’s ability to track metabolic activity and biochemical changes in the media. In the context of neural development, these feedback-enabled systems could offer new insights into how environmental inputs shape dynamic network properties in vitro. Longitudinal, closed-loop experiments may help uncover causal relationships between molecular cues, circuit maturation, and emergent activity patterns, contributing to open questions in developmental neurobiology, such as the timing and regulation of critical periods. Moreover, these capabilities could accelerate disease modeling and drug testing in organoids by allowing precise, state-aware perturbations and measurements.

The interval between electrophysiological recordings is essential for characterizing neural network dynamics. Neural processes unfold with remarkable complexity and variability, but for practical reasons, many experimental paradigms are limited to once-a-day recordings [6,72–74]. Recent work [75] demonstrated that important neural network properties, including firing rate distributions and small-world topology, are “preconfigured” rather than emerging solely through experience-dependent processes. Their finding that stable network properties exist from very early developmental stages validates that automated maintenance and monitoring systems, like the one presented here, can reliably capture intrinsic developmental processes without disrupting natural network organization. By providing the ability to schedule recordings at any interval, our system is particularly well-suited to investigate the relationship between innate and experience-dependent aspects of network development. Monitoring fluctuations across timescales, from minutes to days, aligns with new efforts to understand critical periods of plasticity and how transient network states scaffold long-term function. Our high-frequency recordings revealed trends not captured in once-a-day sampling, enabling the detection of patterns, oscillations, and interactions that may be overlooked in sporadic recordings [76,77]. These capabilities are further relevant for studying phenomena on shorter timescales, such as neuroplasticity [78], circadian rhythms [76], and for investigating neurodevelopmental disorders hypothesized to be ‘connectopathies,’ characterized by abnormal connectivity [79]. By enabling simultaneous tracking of morphological, electrophysiological, and network-level changes over extended time periods, our automated platform could help resolve questions about how early network properties evolve throughout development, potentially yielding new insights into both the stability and plasticity of developing neural circuits. This has implications for not only basic neuroscience, but also for therapeutic discovery and precision medicine, particularly in conditions like epilepsy, autism, and schizophrenia where dynamic changes in network synchrony and excitability are central to pathophysiology.

The greater the complexity of experiments, the more automation becomes essential to coordinate and manage the different technical modalities. The use of 3D printing technology enhances this flexibility, allowing for the seamless combination of multiple systems, such as the integration of our custom media exchange setup with the commercial HD-MEA and portable microscope. We foresee the integration of additional sensory data and feedback mechanisms to analyze cell culture conditions. The lack of effect due to media manipulation presented in Fig. 6 opens the opportunity to dispense and aspirate pharmacological reagents or small molecule factors without the perturbation of manual interventions. With this system one could precisely measure the onset of electrophysiological responses to chemical manipulation of the culture. The platform’s consistency and reliability are ideal for comparative studies involving organoids of different genotypes or subjected to pharmacological manipulations. This capacity to facilitate direct comparisons between diverse experimental conditions in controlled environments holds promise for advancing our understanding of neurodevelopment and neurodevelopmental disorders. Together, these findings demonstrate that automation not only preserves physiological function in this model system but also enables high-resolution, longitudinal insights into neural development, positioning this platform as a foundational tool for scalable and reproducible brain organoid research.

## 4. Materials and methods

### 4.1. Assembled devices and custom 3D-printed components

The Bill of Materials listing components and costs are provided in Supplementary Materials. STL files for 3D printing are provided in PrintedAccessories.zip. All parts were printed using biocompatible resins on a Formlabs Form 3B+ printer with 100 μm layer height, post-processed for 15-min with isopropyl alcohol in the Formlabs Form Wash, and UV cured for 15-min at 60 °C in the Form Cure. The sterilization of the 3D printed parts was completed by a 30 min dry autoclave cycle at 121 °C. See Supplementary Materials section 3D-printed components for more details.

### 4.2. Microfluidic cell culture

The automated microfluidic pump system builds on previous work [63]. The microfluidic system was configured for this study to support two chips (AF and AFAR) and their respective collection reservoirs (right and left) were imaged by the camera setup. Replicates of the conditions were achieved by repeating the experiment on the following batch of organoids.

Fresh cell culture media is kept at 4 °C refrigeration and accessed by the pump through flexible FEP tubing routed into a benchtop refrigerator and to a media bottled with a reagent delivery cap (Cole-Parmer VapLock). Fresh media is kept refrigerated to increase longevity and may be replaced during experimentation. To dispense, the syringe pump and distribution valves draw fresh media into the syringe vial and distribute the programmed volume into flexible FEP tubing routed through an access port in the incubator. Here, the media is heated in incubator conditions prior to being delivered to the organoid inside the culture chamber. To keep media dispenses available on demand, a preheated 450 μL reserve (59% of the chamber’s volumetric capacity) of fresh media remains idle in the FEP tubing so that upon dispensing, 37 °C media is delivered to the well in less than 10 s. The FEP tubing is interfaced with the fluidic module with threaded ferrule lock and nut fittings (Cole-Parmer VapLock). Outflow from the fluidic module is drawn away with FEP tubing routed out of the incubator and into a refrigerator containing the collection reservoirs and computer vision camera setup.

For the collection reservoirs, we selected 15 mL Polyethylene Terephthalate (PET) conical tubes (430 055, Corning) for high optical clarity, ease of replacement, and durability in downstream analysis and cold storage. To enhance visibility for computer vision imaging, we removed the factory-printed writing area on the conical PET tubes using generic, multipurpose tape. Flexible FEP tubing was interfaced with the PET tubes using a rubber cork plug (#6448K95, McMaster-Carr). The cork was pierced with 8-gauge steel needles that served as supportive conduits for the tubing. The tubing was secured inside the needle with glue (Loctite 4011) to create a hermetic seal at the point of interface. The steel encasing of the needles ensures a smooth, unobstructed flow within the flexible FEP tubes. Each collection reservoir had two flexible FEP tubes: one for media coming from the fluidic module and one for pressurized operation connected to the syringe pump. This ensured that spent media never entered the syringe (only air). The air is expelled into a filtered (Millipore AA 0.22 μm syringe filter) safety container (not shown in Fig. 1).

For the 7-day studies described here, we designed for equivalent media exchange across conditions. The Controls were fed 4 times at 1 mL per feed, totaling 4 mL of replacement media. AF and AFAR were fed 28 times at 143 μL per feed, totaling 4 mL of replacement media over the week. Summing the scheduled feeds and feedback adjustments, a single collection reservoir could store conditioned media for 2–3 weeks.

#### 4.2.1. Cell culture

We adapted long-term neural culture approaches compatible with MEA systems to sustain organoid health and network maturation over extended timeframes [80]. Protocols and organoid plating on HD-MEA, which occurred prior to the experiment, are described in Supplementary Materials and Methods.

#### 4.2.2. Priming the experiment

Before starting a 7-day recording experiment, membrane lids for HD-MEAs (AF and AFAR) were replaced with microfluidic culture chambers. During the replacement process, all media was aspirated from the HD-MEA’s well with a P-1000 pipette. The microfluidic catch tray, followed by the culture chamber, was inserted inside the well, and 750 μL of the original media was added back to the microfluidic culture chamber. Excess media was discarded. The glass rod lid was placed on top.

Flexible FEP tubes (idling with DI water) were flushed with 1.0 mL of fresh media. After priming the lines with media, the AF/AFAR chips were connected with fluidic fittings wrapped with Teflon tape. An initial aspiration leveled the media to the target fluidic operating range. The collection reservoirs were replaced with new empty conical tubes.

#### 4.2.3. Running the experiment

During the experiment, the media was exchanged using a feed cycle operation consisting of an aspiration followed by fresh media dispense. Here, we performed 143 μL aspirations and dispenses every 6 h to match 1.0 mL feeds every two days in the manual feeding controls. Feedback performed additional aspiration, dispense, and pull actions in addition to the basic feed cycle schedule to ensure the system stayed within normative error ranges. See section Feedback interpreter. All actions, including the feed cycle, were coordinated and synchronized using our IoT infrastructure based on the MQTT messaging protocol and a Python-based device-class framework, which ensures devices execute scheduled and interdependent tasks without conflict (see Supplementary Materials and Methods sections MQTT and IoT device-class).

#### 4.2.4. Teardown of the experiment

Once the experiment was stopped, chips were disconnected from the flexible FEP tubes by unscrewing the fittings. The flexible FEP tubes with fittings were sterilized in a flask containing disinfectant (Cydex) and covered with aluminum foil. The collection reservoirs with the experiment’s conditioned media were disconnected and taken for analysis. New collection reservoirs were inserted for the cleaning cycle. The pump ran a cleaning solution (Cydex) through the entire internal cavity for 1 h to disinfect the system. Following disinfection, DI water and dry, sterile air were profused through the system for 12+ h (overnight) to clear the disinfectant. The flexible FEP tubes were left resting with DI water until the next experiment.

### 4.3. Computer vision for fluid volume estimation

The Estimator converts captured images into volume estimates using HSV (Hue, Saturation, and Value) segmentation and a calibrated polynomial regression. It distinguishes conical vs. cylindrical regions of the collection tube for accuracy. The result is passed to the pump’s feedback interpreter, which determines corrective actions. The computer vision setup, located inside a 4 °C refrigerator, included a support for the collection reservoir, a camera module, and an LED panel positioned behind the conical tubes. The LED panel served as backlighting to enhance the clarity and contrast of the images. The reservoir support was a two-plex 3D-printed system capable of multiplexity to tailor alternate experiments (see 3D-printed components). The camera and LED panel were both controlled by a Raspberry Pi.

To generate the calibration dataset, the camera module captured images of media in the collection reservoirs at select volumes over the entire range of the tube (0–12 mL), totaling 184 images. The volumes associated with each image were measured using a high-precision scale (30 029 077, Mettler Toledo). This approach enabled a correlation between the visual representation of media in the images and its actual volume (see Results).

To ensure image quality, our study introduced two checks to validate the integrity of the captured images: Lighting and blurriness. A region of interest (ROI) was designated within the panel’s area to verify the lighting conditions by checking that the average RGB color values each exceeded a minimum threshold of 20 out of 255. Blurriness was assessed by computing the variance of the Laplacian for the image, with a necessary threshold of 50 to pass. The thresholds were empirically determined using the calibration dataset.

Fig. 2c illustrates the methodology applied to fluid segmentation, outlined in the Results section. The process begins with capturing an RGB image of the collection reservoirs that are fixed in place by the setup. To facilitate better segmentation and feature extraction, the RGB image is transformed into the HSV (Hue, Saturation, and Value) color space. A summation of the HSV values row-wise from the bottom to the top of the collection reservoir results in three distinctive profiles that allow differentiation between the liquid and background. Each profile, as illustrated in Fig. 2c, presents a vertex at the boundary. A row value was established by averaging three rows identified in each HSV channel: an abrupt rise in the curve for the Hue channel, the absolute maximum for the Saturation channel, and the absolute minimum for the Value channel. From the average row value, the first segmentation was created. Everything below this row was set as white pixels, and everything above it was set as black pixels. A local evaluation around the average row was made to incorporate the meniscus in this segmentation. Utilizing HSV thresholds, the meniscus was accurately characterized and incorporated into the initial segmentation, culminating in the final image segmentation, in which white pixels represented the liquid portion.

The estimated volume was given by Eq. (1), where x represents the segmented area in pixels, and the resultant volume is in microliters. Two different curves are used to account for the conical section for volumes under 1.5 mL (and pixel area less than 4446) and the cylindrical section for larger volumes.

(1) $$V\left(x\right)=\{\begin{array}{ll} 5.09\times 10^{-9}x^{3}+2.39\times 10^{-5}x^{2}+0.13x-1.28 & \text{if}\;x<4446\;\text{pixels}\\ 2.60\times 10^{-11}x^{3}+5.38\times 10^{-7}x^{2}+0.62x-1288.37 & x\geq 4446\;\text{pixels} \end{array}$$

The image segmentation and estimation based on the mathematical model (Eq. (1)) is carried out by a software program named the “Estimator”. The process initiates with a feeding cycle, which triggers a picture request. Upon receiving the image of the collection reservoir, the “Estimator” analyzes the image and returns the estimated value of the fluid volume. The volume is relayed to the next module for feedback interpretation within the pump system (see Feedback interpreter).

### 4.4. Feedback interpreter

Computer vision volume estimations were compared to expectation values based on the sum total of pump action jobs. The feedback interpreter classified estimations into four categories: within tolerance, out-of-tolerance, anomaly, and tube change. Tolerance was a static volume selected at the start of the experiment. For the results shown here, the tolerance was 143 μL. If the volume estimation received was within the expectation value ± the tolerance, the pump action was determined a success, and feedback ceased. If the volume estimation received was beyond the expectation value ± the tolerance and also less than ±2000 μL, another cycle of feedback was engaged. When the volume was less than expected, for the first 5 iterations of feedback, aspiration jobs were sent to the pump with the difference of expectation and estimation. For iterations 6 to 19, pull jobs were sent to the pump, increasing by one for each subsequent interaction. A “pull” is a 1000 μL aspiration at 10× the standard syringe speed (applying a 1.1 × 103 mm/s flow rate), shown to generate the force required to break through variably high resistance in the conditioned media. At 20 iterations, the feedback interpreter requests manual intervention via the messaging application, and all further pump actions are suspended until the issue is resolved. When the volume was more than expected, dispense jobs were sent to the pump with the difference of expectation and estimation. Dispense actions were limited to 200 μL per action and 2 iterations of feedback in total to prevent overflow. A volume estimation that was 2000 μL or more above the expectation value was determined as an anomaly and requested manual intervention via the messaging application, and all further pump actions were suspended until the issue was resolved. The feedback interpreter automatically detected collection reservoir tube changes when the volume estimation dropped by 2000 μL or more compared to the previous estimation and the total volume present was estimated as less than 2000 μL.

### 4.5. Computer vision for in-incubator organoid culture imaging

#### 4.5.1. In-incubator imaging

A 5MP digital microscope (AM7115MZTL, Dino-Lite) was placed over the organoid culture on the HD-MEA using holders described in Assembled devices and custom 3D printed components. Imaging was performed from the top through a glass rod (quartz drawn rod, 5 mm ± 0.20 mm dia × 15 mm ± 0.20 mm long, UQG Optics) (in AF/AFAR chips) or through a membrane lid (in control chips). The image is captured using reflected light from a built-in brightfield LED source next to the camera sensor. The 3D printed alignment trays handle most of the chip placement, with initial minor focal plane adjustment required. The microscope remains shut off until the software triggers it to turn on the lights and take a photo.

#### 4.5.2. Image segmentation for organoid

In the process of image segmentation for organoid analysis, the first step involves applying an image calibration to correct any distortion. This procedure requires identifying four source points and four destination points. The former were manually selected from the distorted image. The latter were calculated based on an initial pixel (left corner of the HD-MEA), the size of the electrodes, and the spacing between them, both in millimeter units. This relationship between pixels and millimeters was established by using known dimensions of the HD-MEA border and electrode pitch in the image.

The organoid segmentation within the rectified image was accomplished using the Segment Anything Model (SAM) [81]. This model combines neural network architectures, allowing for precise and versatile image segmentation without requiring specialized training on new images. The segmented image is analyzed to detect variations in pixel intensity, which signify the presence of organoid contours. Both images with the organoid’s contour and electrode grid are overlayed. Each electrode area is checked for the presence of the organoid’s border. When a border is detected within an electrode’s bounds, that particular electrode is marked prominently on the grid image to signify contact with the organoid (see Fig. 5b). The step-by-step illustration of the analysis process is shown in Supplemental Figure 4.

#### 4.5.3. Plotting & alignment to neural activity data

Electrode numbers as (x,y) position were plotted in matplotlib and exported as SVG. The SVG aligns over other plots, such as activity heatmaps, which follow the same x:3580 by y:2100 axis dimensions. Since electrophysiology plots use the electrode coordinate system with the same (x,y) positions, the image segmentation grid and neural activity plots are aligned on the same coordinate system.

### 4.6. Measuring neural activity

Extracellular field potential recordings were performed using CMOS-based high-density microelectrode arrays (HD-MEAs) (MaxOne, Maxwell Biosystems). Each HD-MEA contains 26,400 recording electrodes within a sensing area of 3.85 mm × 2.1 mm (each electrode has a diameter of 7.5 μm, spaced 17.5 μm apart center-to-center). A subset of up to 1020 electrodes (defined spatially by a configuration) can be selected for simultaneous recording [82]. Across one configuration, neuronal activity in microvolts was sampled over time at 20 kHz and stored in HDF5 file format.

The experiment involved each chip’s daily activity scans and recordings (described below). Each chip underwent an activity scan and subsequent recording every day, consistently conducted within the same one-hour time window. All chips shared the same recording unit and were recorded one at a time. For the AFAR condition, beyond the daily recordings and activity scans, the chip remained on the HD-MEA for automated hourly recordings. To ensure reliability of spike sorting and neural signal analysis, we applied quality control metrics commonly used in extracellular recordings [83]. The gain was set to 1024× with a 1 Hz high pass filter for both activity scans and recordings. The recording was set up to save 5 RMS thresholded spike times as well as all raw voltage data for downstream analysis and plotting.

All neural activity measurements were performed inside the incubator at 36.5 °C, 5% CO2.

### 4.7. Normalization and z-score calculation for effect of feeding during recording

In section Effects of feeding during recording, Fig. 6d, the firing rate variability to feeding was derived as follows:

For every recording, a 90-s sliding window was used to further analyze the sorted and curated spike raster with steps of 1-s. For every window, the average firing rate per unit was computed. To account for intrinsic variability in firing rate between units, the average firing rate per window was normalized for each unit as ‖x‖=(a−m)/(a+m), where ‖x‖ is the norm, *a* is the window rate and *m* is the mean value across all window rates in the recording. This normalization yields a values between −1 and 1, reflecting the deviation from the average firing rate of each unit. A value of 0 means that the average firing rate for a unit in a given window is the same as the average firing rate for that unit. A value of ±1/3 means that the firing rate for a unit in a given window is approximately twice (for +1/3) or half (for −1/3) the mean firing rate for that unit.

Subsequently, the normalized firing rates per unit for all no-feed recordings were taken and for each window relative to the start of the recording, the mean and standard deviation were computed over all units in all no-feed recordings. These values were then used to z-score normalize the normalized firing rates of every unit in every window throughout all feed recordings as $z=\left(\left\|x_{feed}\right\|-\mu_{nofeed}\right)/\sigma_{nofeed}$, where *z* is the z-score, $\left\|x_{feed}\right\|$ is the norm in the feeding condition, $\mu_{nofeed}$ is the mean of the norm in the no feeding condition, and $\sigma_{nofeed}$ is the standard deviation of the norm in the no feeding condition. This normalization yields a value for each unit per fed recording at every window that reflects the variability from the average firing rate of that unit. This is expressed as the number of standard deviations that any particular value is removed from the average variability from the mean firing rate of all units over all no-feed recordings at that same window in the recording. If the firing rate of the unit during any point in the feed recording substantially increases or decreases relative to the average firing rate over the whole recording and if this is not the case for units in the no-feed recording, this will be reflected as a positive or negative z-score normalized value. In addition, the z-scores were averaged over all units in all feed recordings with the same feed volume to see the effects for each individual feed volume.

### 4.8. Internet of Things (IoT)

#### 4.8.1. Cloud infrastructure

The cloud infrastructure, including S3, MQTT messaging, and cloud processing within the IoT system, has been previously described [59]. To streamline team communication and real-time monitoring, we integrated messaging with our automation pipeline [84]. Additionally, we added a database service and defined a consistent organizational structure for MQTT messages and topics across devices and cloud jobs.

We use a combination of self-hosted services running on a server, and large data storage and analysis are performed on the National Research Platform (NRP) cloud compute cluster [85]. The devices are integrated with these cloud services:

- S3 cloud data storage: file storage using S3 object store, hosted on NRP cloud.
- Database: Strapi database stores device states, is self-hosted on our server, and is backed up to S3.
- MQTT messaging: EMQX MQTT broker, self-hosted on the server, and a Python messaging library (braingeneers.iot.broker) utilized by all software endpoints to send and receive messages from the broker.
- Cloud jobs/processing: utilizes a Kubernetes cluster on NRP and launches jobs. Employs software modularized by Docker containers and orchestrated by Kubernetes.
- User interfaces: features a website and integration with messaging apps (e.g., Slack) for interaction with devices, self-hosted on the server.

All custom software functionalities run in Docker containers and operate in a microservice architecture: specialized to a specific task and interface with minimal dependencies. A reverse proxy shields all web services from direct exposure to the internet. For example, webpages are configured through a reverse NGINX proxy, which not only assigns a specific domain to each service but also handles SSL and authentication services.

#### 4.8.2. Security

Devices initiate communication with the server. Devices take MQTT commands in a specific format and are limited to the set of their defined commands, making them robust to command injection attacks. Accessing all cloud services requires authentication with credentials. All web, MQTT messages, database, and S3 storage operations are encrypted. Access to the user interface website is restricted through the proxy with a login authentication step. On the server side, all web-based microservices are secured through an NGINX proxy. The proxy allows web-based services to be relatively untrusted by providing security (https, authentication, internet visible network listener) and keeping all other web-based services on an internal docker network inaccessible from the internet. This simplifies security for services that may change often and accommodates programmers with minimal security training.

## Supplementary Material

Supp.MatMethod

SuppMat2

## Data Availability

Electrophysiological data are hosted on a DANDI public server: https://dandiarchive.org/dandiset/001268. Other data including Bill of Materials, CAD models, are available in GitHub: https://github.com/braingeneers/feedback-organoid-platform.

## Appendix A. Supplementary data

Supplementary material related to this article can be found online at https://doi.org/10.1016/j.iot.2025.101671.
