# Supplementary material for: A feedback-driven brain organoid platform enables automated maintenance and high-resolution neural activity monitoring
Source: Internet Things (Amst). Author manuscript; Available in PMC 2026 Jan 9. (PMC12781996; doi:10.1016/j.iot.2025.101671)
Supplement: Supp.MatMethod [file NIHMS2123043-supplement-Supp_MatMethod.pdf]

Supplementary Materials and Methods

Embryonic stem cell culture

All experiments were performed in the adapted C57/BL6 mouse embryonic stem cell (ESC) line (Millipore Sigma # SF-CMTI-2). This line is derived from a male of the C57/BL6J mouse strain. Mycoplasma testing confirmed lack of contamination.

ESCs were maintained on Recombinant Human Protein Vitronectin (Thermo Fisher Scientific # A14700) coated plates using mESC maintenance media containing Glasgow Minimum Essential Medium (Thermo Fisher Scientific # 11710035), Embryonic Stem Cell-Qualified Fetal Bovine Serum (Thermo Fisher Scientific # 10439001), 0.1 mM MEM Non-Essential Amino Acids (Thermo Fisher Scientific # 11140050), 1 mM Sodium Pyruvate (Millipore Sigma # S8636), 2 mM Glutamax supplement (Thermo Fisher Scientific # 35050061), 0.1 mM 2-Mercaptoethanol (Millipore Sigma # M3148), and 0.05 mg/ml Primocin (Invitrogen # ant-pm-05). mESC maintenance media was supplemented with 1,000 units/mL of Recombinant Mouse Leukemia Inhibitory Factor (Millipore Sigma # ESG1107). Media was changed daily.

Vitronectin coating was incubated for 15 min at a concentration of 0.5 µg/mL dissolved in 1X Phosphate-buffered saline (PBS) pH 7.4 (Thermo Fisher Scientific # 70011044). Dissociation and cell passages were done using ReLeSR passaging reagent (Stem Cell Technologies # 05872) according to the manufacturer's instructions. Cell freezing was done in mFreSR cryopreservation medium (Stem Cell Technologies # 05855) according to the manufacturer's instructions.

Cerebral cortex organoids generation

Mouse cortical organoids were grown as previously described by our group [46, 15] with some modifications. To generate cortical organoids we single cell dissociated ESCs using TrypLE Express Enzyme (ThermoFisher Scientific #12604021) for 5 minutes at 37°C and re-aggregated in lipidure-coated 96-well V-bottom plates at a density of 3,000 cells per aggregate, in 150 µL of mESC maintenance media supplemented with Rho Kinase Inhibitor (Y-27632, 10 µM, Tocris # 1254) and 1,000 units/mL of Recombinant Mouse Leukemia Inhibitory Factor (Millipore Sigma # ESG1107) (Day -1).

After one day (Day 0), we replaced the medium with cortical differentiation medium containing Glasgow Minimum Essential Medium (Thermo Fisher Scientific # 11710035), 10% Knockout Serum Replacement (Thermo Fisher Scientific # 10828028), 0.1 mM MEM Non-Essential Amino Acids (Thermo Fisher Scientific # 11140050), 1 mM Sodium Pyruvate (Millipore Sigma # S8636), 2 mM Glutamax supplement (Thermo Fisher Scientific # 35050061) 0.1 mM 2-Mercaptoethanol (Millipore Sigma # M3148) and 0.05 mg/ml Primocin (Invitrogen # ant-pm-05). Cortical differentiation medium was supplemented with Rho Kinase Inhibitor (Y-27632, 20 µM # 1254), WNT inhibitor (IWR1- ε , 3 µM, Cayman Chemical # 13659) and TGF-Beta inhibitor (SB431542, Tocris # 1614, 5 µM, days 0-7). Media was changed daily.

On day 5, organoids were transferred to ultra-low adhesion plates (Millipore Sigma # CLS3471) where media was aspirated and replaced with fresh neuronal differentiation media. The plate with organoids was put on an orbital shaker at 60 revolutions per minute. Neuronal differentiation medium contained Dulbecco's Modified Eagle Medium: Nutrient

Mixture F-12 with GlutaMAX supplement (Thermo Fisher Scientific # 10565018), 1X N-2 Supplement (Thermo Fisher Scientific # 17502048), 1X Chemically Defined Lipid Concentrate (Thermo Fisher Scientific # 11905031) and 0.05 mg/ml Primocin (Invitrogen # ant-pm-05). Organoids were grown under 5% CO2 conditions. The medium was changed every 2-3 days.

On day 14 and onward, we transferred the organoids to neuronal maturation media containing BrainPhys Neuronal Medium (Stem Cell Technologies # 05790), 1X N-2 Supplement, 1X Chemically Defined Lipid Concentrate (Thermo Fisher Scientific # 11905031), 1X B-27 Supplement (Thermo Fisher Scientific # 17504044), 0.05 mg/ml Primocin (Invitrogen # ant-pm-05) and 0.5% v/v Matrigel Growth Factor Reduced (GFR) Basement Membrane Matrix, LDEV-free.

Organoid plating on microelectrode array

Mouse cerebral cortex organoids were plated, as previously described by our group [15], with two organoids per well. We plated the organoids at day 32 on MaxOne high-density microelectrode arrays (Maxwell Biosystems # PSM). Prior to organoid plating, the microelectrode arrays were coated in 2 steps: First, they were coated with 0.01% Poly-L-ornithine (Millipore Sigma # P4957) at 36.5°C overnight. Then, the microelectrode arrays were washed 3 times with PBS and coated with a solution of 5 µg/ml mouse Laminin (Fisher Scientific # CB40232) and 5 µg/ml human Fibronectin (Fisher Scientific # CB40008) prepared in PBS, at 36.5°C overnight.

After coating, we placed the organoids on the microelectrode arrays and removed excess media. The organoids were incubated at 36.5°C for 20 minutes to promote attachment. We then added prewarmed neuronal maturation media (described in the section above). We exchanged 1.0 mL of conditioned media for fresh every 2 days.

HD-MEAs containing the organoid cultures are stored in an incubator at 36.5 °C, 5% CO2, covered with membrane lids described in the section below, 3D-printed components.

Computer vision for fluid level detection

Camera details

A 16MP camera (B0290, Arducam) and a set of conical tubes are fixed 12 mm apart from each other on an optical breadboard (SAB10x30-M, Base Lab Tools). The camera was specifically configured without autofocus, with its focus statically set at 344 on a scale from 1 to 1023. A two-second warm-up period stabilizes the focus setting before a picture is taken. Exposure was set at 45 on a scale from 1 to 5000.

LED panel details

A 16x16 LED matrix (WS2812B-16x16ECO, BTF-LIGHTING) covered with 0.1mm thick polyester diffusion film (B08PTCGTX9, RENIAN) creates a uniformly illuminated background (we used 8 sheets of diffuser film spaced 1 mm apart by double-sided foam mounting tape). The LED panel is approximately 5 mm behind the conical tubes.

The LED matrix was set to display a color gradient to best contrast fluid contents inside the conical tube, particularly in the cone-shaped lower area of the conical tube, which is thinner and appears lighter in color. The red color component of each LED matrix pixel was set based on its row position within the matrix, beginning with an initial red value of 221

out of 255. The red color intensity was reduced by two units for each row upwards, creating a gradient effect. Thus, the final color of each pixel was a combination of this dynamically adjusted red value and fixed green and blue values of 140 and 180, respectively. Furthermore, the LED panel's brightness was set to 50% to prevent overexposure in the captured images.

3D-printed components

All custom accessories were 3D printed (Form 3B+, Formlabs) with Biomed Clear V1 material (RS-F2-BMCL-01, Formlabs), except for the collection tube and camera stand in the refrigerator printed in BioMed Black V1 (RS-F2-BMBL-01, Formlabs). The parts were printed flat on the build plate to reduce support material. Alignment grooves between the insert and lid described in the Microfluidic culture chamber form a hole which also facilitates 3D printing by removing the formation of suction cups to the resin tank.

Microfluidic culture chamber

The microfluidic culture chamber assembly allows media to be exchanged inside the HD-MEA well. The chamber assembly consists of a microfluidic module, glass rod lid, and catch tray (Figure 1b,f,g).

The microfluidic module is placed inside the HD-MEA well, creating a media chamber and fluid path into and out of the chamber. Media from outside the incubator travels to the fluidic insert along 0.030" ID and 0.090" OD Tygon tubing (AAD02119-CP, Cole Parmer); the length of the tubing is approximately 100 cm. The tubing attaches to the fluidic insert using PEEK fittings (EW-02014-97, Cole Parmer) wrapped (counter-clockwise) in PTFE thread seal tape around twice the fitting's circumference. The inlet and outlet are raised inside the fluidic insert to create a pool following a geometry published in previous work (30).

The fluidic insert, glass rod lid, and catch tray use silicone O-rings (5233T543, 5233T479, 5233T297, and 5233T585, McMaster) to provide seals against contaminations and leakage. O-rings were rubbed with a minimal quantity of canola oil for lubrication to facilitate installation and enhance sealing performance. The canola oil can be autoclave-sterilized, but it is unnecessary if the O-rings are sterilized post-lubrication (see section, Sterilization and assembly).

Membrane lid

The membrane lid used for experimental control conditions follows established designs (49), with adjusted dimensions to improve grip, matching material to the microfluidic culture chamber, and high-temperature silicone O-rings instead of rubber. The outer O-ring (5233T683, McMaster) holds the breathable FEP film (23-1FEP-2-50, CS Hyde Company) stretched over the top of the lid. The inner O-ring (5233T585, McMaster) seals the lid and well. The inner O-ring is also rubbed with a minimal quantity of canola oil as described in the Microfluidic culture chamber section.

In-incubator imaging alignment holders

The custom alignment holders, designed for two configurations, center a digital microscope over the biological sample on the HD-MEA. Components are screw mounted (91292A134, McMaster) to optical breadboards (SAB10X15-M, SAB15X15-M, Base Lab Tools Inc.) to ensure stability and maintain accurate spacing.

HD-MEA off the recording unit

The microscope is held over a single HD-MEA by a post and clamp (MS08B, Dino-Lite) mounted with a setscrew and base (SS6MS10, TH15/M, Thorlabs). The custom HD-MEA holder centers it for imaging. Throughout the experiment, HD-MEAs were left resting on each holder. The holder has cut-outs for handling the chip and also avoids the chip's contact pads to decrease scratching and avoid moist surfaces. The holder also has indicators for the chip's proper rotation with respect to the microscope.

HD-MEA on the recording recording unit

The custom holder on a post assembly (SS6MS10, TH15/M, TR250/M-JP, Thorlabs) mounts the microscope over the chip on the recording unit. The custom holder centers both the recording unit with its attached chip to the microscope.

Sterilization and assembly

Before use in tissue culture, components were placed in autoclavable bags (RIT-3565, PlastCare USA) and steam-sterilized at 134°C for 20 minutes or 121°C for 30 minutes based on Formlabs material datasheet specifications. Components were autoclaved, disassembled, and then assembled in a sterile tissue culture hood to avoid deformation or cracking during temperature cycling. Components were transported in an enclosed petri dish (small items) or a sterile autoclaved bag (large items) before being released into the incubator. Components that could not be autoclaved (such as electronics, i.e., recording unit, microscope) have their enclosures sterilized with hydrogen peroxide disinfecting wipes (100850922, Diversey) before entering the incubator.

Measuring neural activity

Activity scans

Activity scans were performed daily in the MaxLab Live Scope (Version 22.2.22, MaxWell Biosystems) to identify where the organoid's electrical activity is spatially distributed across the HD-MEA. The activity scan sequentially records from different configurations of up to 1020 electrodes, thereby sampling the microelectrode array for action potentials. We used the checkerboard assay consisting of 14 configurations, with 30 seconds of recording per configuration. The resulting activity heatmap (see Activity heatmaps) for each chip is shown in Figure 5b. Based on the assay results, 1020 most active electrodes were selected for simultaneous activity recordings.

Recordings

Each recording lasted 10 minutes. Initial recording configurations were created on the first day, and configurations were updated on the second day to match shifting activity. Afterward, we chose to keep the configurations constant across the final 5 days since the activity did not shift dramatically, and keeping the same configuration allowed for more consistent monitoring of the same region.

Smartplugs

A smartplug was connected to the recording system to automatically manage the duration of the recording system running. The smartplug (S31, SONOFF) running Tasmota 13.2.0

was connected to the MQTT broker (see MQTT) and received MQTT commands over WiFi to turn on and off.

The smartplug facilitated the automated recordings every hour: on the computer connected to the MEA recording system, a script running in Python (3.10) triggered the smartplug via MQTT to turn on the recording system, performed a recording using MaxLab Python API (MaxWell Biosystems), and afterward triggered the smartplug to turn off the recording system.

Spike sorting and curation

To process the electrophysiology data, each MaxWell recording was spike sorted into single unit activity using Kilosort2 [44]. Using a template-matching algorithm, Kilosort2 clustered neurons based on waveform shape. Spike sorting parameters included a bandpass filter of 300 to 6000 Hz for the raw data and voltage threshold of 6 RMS above baseline.

The sorting output was curated by an automatic algorithm that quality checks signal-to-noise ratio (SNR), firing rate, interspike interval (ISI) violation, and spike footprint for each putative neuronal unit. As a result, units that had SNR above 3, firing rate above 0.1 Hz, ISI violation below 0.5 and footprint on more than one channel were kept for analysis [19]. Units were labeled redundant using spikeinterface "remove_redundant" module and processed through manual curation for consolidation.

Spike sorting was performed on the National Research Platform (NRP) computing cluster with an NVIDIA A10 GPU.

Activity heatmaps

Activity heatmaps in Figure 5a depict the spatial distribution of significant voltage events. MaxWell software provides thresholded event identification based on moving root-mean-square (rms) value for each electrode, identifying events exceeding 5 times an electrode's rms value. We created a 2D grid of spike counts per second and applied a 2D Gaussian blur for visual smoothness, normalizing each grid point by dividing it by $2\pi r^2$ to re-scale back to the original Hz values. These values were then plotted as the activity heat maps. The heatmaps use warmer colors for higher firing frequency and darker colors for lower activity.

MQTT

MQTT messages serve as the standard unit of communication (Figure 3b, orange). MQTT allows devices and services to communicate without direct dependencies between each other by using a common publish/subscribe medium. MQTT clients are the devices or software entities that connect to the broker to send (publish) or receive (subscribe to) messages. Devices and services send messages on MQTT topics, which are hierarchical strings that allow listeners to capture a wide or narrow scope of information. Messages contain a payload with a list of key-value pairs to structure information. For example, a message requesting a microelectrode array to record has a key for recording duration with a value in minutes. Examples of MQTT topic structure and message JSON payloads are summarized in Supplementary Table 1; see GitHub for more information[1].

[1] `https://github.com/braingeneers/integrated-system-v1-paper`

The MQTT broker is the central communication facilitator in the network and coordinates messages between clients. The MQTT broker receives all messages from the clients, filters these messages based on their topics, and then distributes them accordingly to other clients who have subscribed to those specific topics. This setup enables efficient message routing and ensures that messages reach the intended recipients without the senders needing to know the specific details of the recipients.

Clients can be sensors, actuators, applications, and services (like UIs or analysis), or any other devices capable of network communication. The organization is future-proof because MQTT allows the creation of new services and devices and uses information available without changing any services (logging, UI, dashboards, analysis of traffic, etc.). Furthermore, message bridges can be employed to convert MQTT messages to other messaging APIs such as text messaging, email, or work chat applications like Slack (see Messaging bridge).

IoT device-class

The primary function of a *device-class* involves listening for job requests, executing them, and saving the resulting data to the cloud. This data includes measurements (e.g., images, voltage recordings) and log entries detailing device actions (e.g., cell culture feeding events). By consolidating features, the *device-class* framework simplifies the creation of new devices and enables easy control, updating, and interoperability. The Python *device-class* provides standard features across all IoT devices:

- a state machine defining standard behavior (i.e., experiment workflow)
- structured framework for processing incoming request messages
- autonomous task scheduling, timing, and execution; the internal scheduler manages time
- conflicts of tasks or autonomously recurring jobs
- multi-tasking and responsiveness to user requests via threading
- built-in database operations (i.e., updating device state (shadow))
- communication via MQTT messaging (including alerts via Slack bridge)
- background data upload/download mechanisms, managing queueing and retry
- error handling mechanisms
- communicate and work with other devices in a fleet

A child of the parent *device-class* will inherit all basic functionality, and may add additional features. For instance, a camera *device-class* child performs all actions that a *device-class* can, plus it knows how to handle a request to take a picture.

Having a common parent class consolidates similar features for different devices and allows for easier updates because all devices use the same core code library. The *device-class*

code is available within the Braingeneerspy Python package on GitHub[2]. For state machine states and request commands see Supplementary Tables 1 and 1.

Devices can work in a fleet. As each device has the same core software with complementary behaviors, they integrate seamlessly, similar to how uniform building blocks can easily snap together. Devices can ask each other to yield while they perform sensitive actions (Figure 3d). Similarly, devices can perform services for each other in a coordinated manner. For example, midway through a recording, a microelectrode array device could ask the pump to deliver a drug. Devices can perform rudimentary decision-making to simplify overarching management. Devices post status and information to an open MQTT topic, allowing services and devices to build on and interface with those devices without altering existing devices and services. Devices can use each other to make sure the experiment is on track across multiple modes of sensing, for example the pump using the eyes of the camera to ensure pumping succeeded.

Pre-experiment workflow

Figure 3a illustrates the state transitions of a generic device during operation. It begins in the SHUTDOWN state, moving to IDLE, where it waits for user setup verification. Post-setup, it transitions to PRIMED, ready for experimental involvement. In the READY state, the device listens for experiment-specific MQTT messages, ignoring external recruitment until released with an END message. Devices can communicate collectively via MQTT topics for coordinated actions. Transitioning to WAITING occurs upon receiving a pause command, halting job execution. The device moves to EXEC when starting a job, returning to READY upon completion. Data uploads are managed independently of state changes, ensuring continuity even during outages. Devices can exit an experiment at any stage, reverting to IDLE or SHUTDOWN, with data upload tasks resuming upon restart. Figure 3a describes a generic device (e.g. a scientific instrument) and how it transitions between states during operation. On device start, the device transitions from SHUTDOWN state to IDLE. In the IDLE state, the device is waiting for a user to verify or install physical prerequisites. The IDLE state ensures the user performs the necessary setup of their device to maintain safety and usability. For example, a pump may wait in IDLE state until a user checks and confirms that the pump is clean and proper reagent bottles are connected. On the other hand, a camera may not have any prerequisites and would immediately transition to the next state, PRIMED. In the PRIMED state, the device has all the prerequisites to perform its job and waits to be called into an experiment. Devices listen on their default device MQTT topic. Once it receives a correctly formatted 'start' MQTT message (see 'START' message in Supplementary Table 1), it can transition to READY.

Experimental workflow

When the device transitions to READY state, when it listens to an MQTT topic for the experiment. It will refuse requests to be recruited to other experiments until it is released from the current experiment by an END message (see END message in Table 1). This ensures other users don't accidentally disturb or recruit an occupied device into a parallel experiment. Switching MQTT topics also ensures exclusivity in incoming messages. The

[2] `https://github.com/braingeneers/braingeneerspy`

experiment topic structure (see MQTT) allows devices to send a group message addressing all devices. For example, a device or user could send a message to roll-call all devices on the topic (see PING message in Table 1) or pause all devices while it performs a sensitive action (see PAUSE message in Table 1). Upon receiving a message to pause, the device transitions to WAITING state, where it does not perform any jobs.

Once a device returns to READY state, it can transition into EXEC state if it receives a job request or has a job request from its schedule. If the device is in WAITING or EXEC while receiving a job request, it will put the request on the schedule to be executed as soon as possible. During EXEC state, the device is actively executing a job request. Once the job finishes or is stopped (see STOP message in Table 1), the device transitions back to READY state. Any data produced is queued for upload, protected from internet outages by upload retries with exponential backoff. Uploads occur in the background, independent of device state. A device can begin EXEC on a new job immediately after queueing the previous data for upload. From any state, a device can be terminated from an experiment and return to the IDLE state. At any point in the experiment, if a device is gracefully requested to turn off, it performs a final transition to SHUTDOWN state before halting the program. The device keeps the upload queue saved on disk and will continue unfinished uploads upon restart.

Data uploading

Data is saved to a 'diskcache' in memory. Once a file is produced, it is put on the upload queue. The upload queue contains references to files within diskcahe. Typical devices have at least 32 GB of disk memory, far larger than a single file. The queue is restricted to grow up to 80% of the device's memory. Once the memory of the device fills up, older files that were uploaded can become overwritten.

Messaging bridge

The messaging bridge serves as an intermediary for communication between different platforms. It is a service that listens to MQTT messages in the IoT environment and translates them into other APIs like Slack.

The Slack bridge allows IoT devices to send notifications to individuals in designated Slack channels. The messaging bridge uses the message broker API and Slack API [67]. The Slack API requires an API key to be registered with Slack and an API bot to be added to the Slack channels of interest. The message bridge listens to an MQTT channel dedicated to Slack messages. When devices want to post a message to Slack, they publish a message on the dedicated Slack MQTT topic with a JSON payload containing the message. The payload can include text and image data. To support images, a link to an S3 object can be passed in the message, and the messaging bridge will then download and attach it to the Slack message. An image can also be sent directly inside the MQTT message, this requires modifying the message broker service's configuration to increase the MQTT message buffer size to accommodate larger KB-sized files. The Slack bridge is a relatively simple service that decouples devices from dependencies on a specific API by communicating using the common message format MQTT.

Website

The website's front end is developed using React, a JavaScript library for building dynamic and responsive user interfaces. For the backend, Flask, a lightweight Python web framework, is employed. Flask's simplicity and flexibility make it ideal for our web services. It handles server-side operations, data processing, and interaction with databases.

The system's structure incorporates a message broker API, which is established on the backend side of the architecture. This message broker is responsible for the asynchronous communication and management of all IoT devices connected to the cloud. Additionally, Flask's compatibility with Python enables seamless integration with Python APIs, including the braingeneerspy MQTT message broker.

Through the front end, users can issue commands to the devices, and the message broker API in the backend efficiently manages these requests. The user interface encompasses three main components: the initialization page for entering initial experiment data, the control page for managing devices and monitoring their status, and the visualization page for analyzing experimental data through various graphs. All three pages require a specified experiment UUID (see Figure 3).

Both frontend and backend components are containerized using Docker, ensuring consistency and isolation in different environments. Integration of Cross-Origin Resource Sharing (CORS) is crucial for allowing the React frontend to securely interact with the Flask backend hosted on a different domain.

Initialization page: On the initialization page, users can enter metadata containing experiment and biological sample details, which are compiled into a JSON file and uploaded to cloud storage, serving as a centralized repository for all experimental data.

Control page: On the control page, users can access all the devices involved in the experiment associated with a specific UUID. For each device, users can request the execution of all the commands listed in Table 1, such as starting, stopping, and pausing the device, as well as scheduling tasks. Additionally, on the control page, users can monitor the real-time status of the device, as outlined in Table 1.

Visualization page: On the visualization page, users can load data related to the volume estimator from current or previous experiments of a specific UUID. It is also possible to download images on a specific timestamp, allowing for manual monitoring of reservoir tubes.

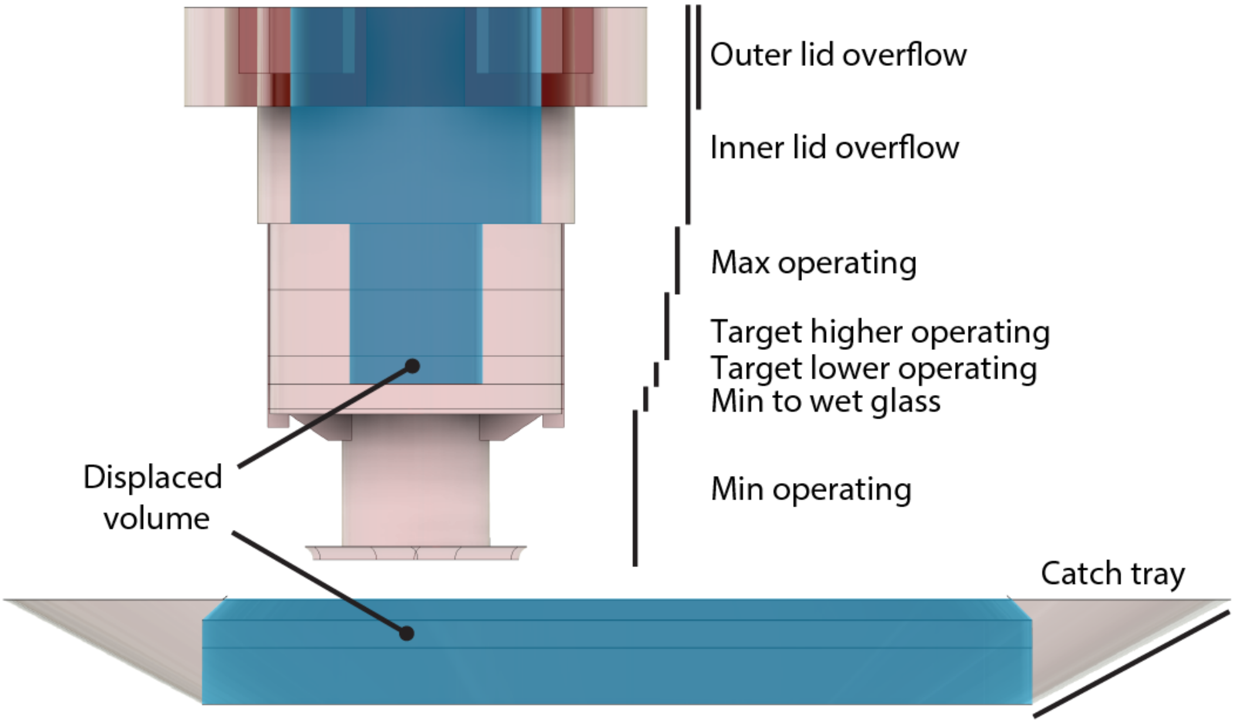

Supplementary Fig. 1: **Diagram of operating ranges of the microfluidic culture chamber.** Shaded pink areas represent volumes where media is collected. Shaded blue areas mark displaced volumes (where there is no media stored). The numerical volumes for each operating range are listed in Supplementary Table 1.

**Feature**	**Delta (µL)**	**Running Total (µL)**
Min operating	172.2	172.2
Min to wet glass	94.0	266.2
Target lower operating	88.1	354.3
Target higher operating	204.9	559.2
Max operating	204.9	764.1
**Total operating capacity:**		**764.1**
Inner lid overflow	345.1	1109.2
Outer lid overflow	464.1	1573.3
**Total chip capacity:**		**1573.3**
Catch tray	1539.5	3112.8
**Total overflowed capacity:**		**3112.8**

Supplementary Table. 1: Numerical operating volume ranges based on the microfluidic culture chamber's 3D model (CAD) measurements. Illustrations of operating ranges are shown in Supplementary Figure 1. The Feature column lists critical points in the microfluidic culture chamber. The Delta column is the volume space between each feature, and the Running Total column is the volume from the floor to the feature.

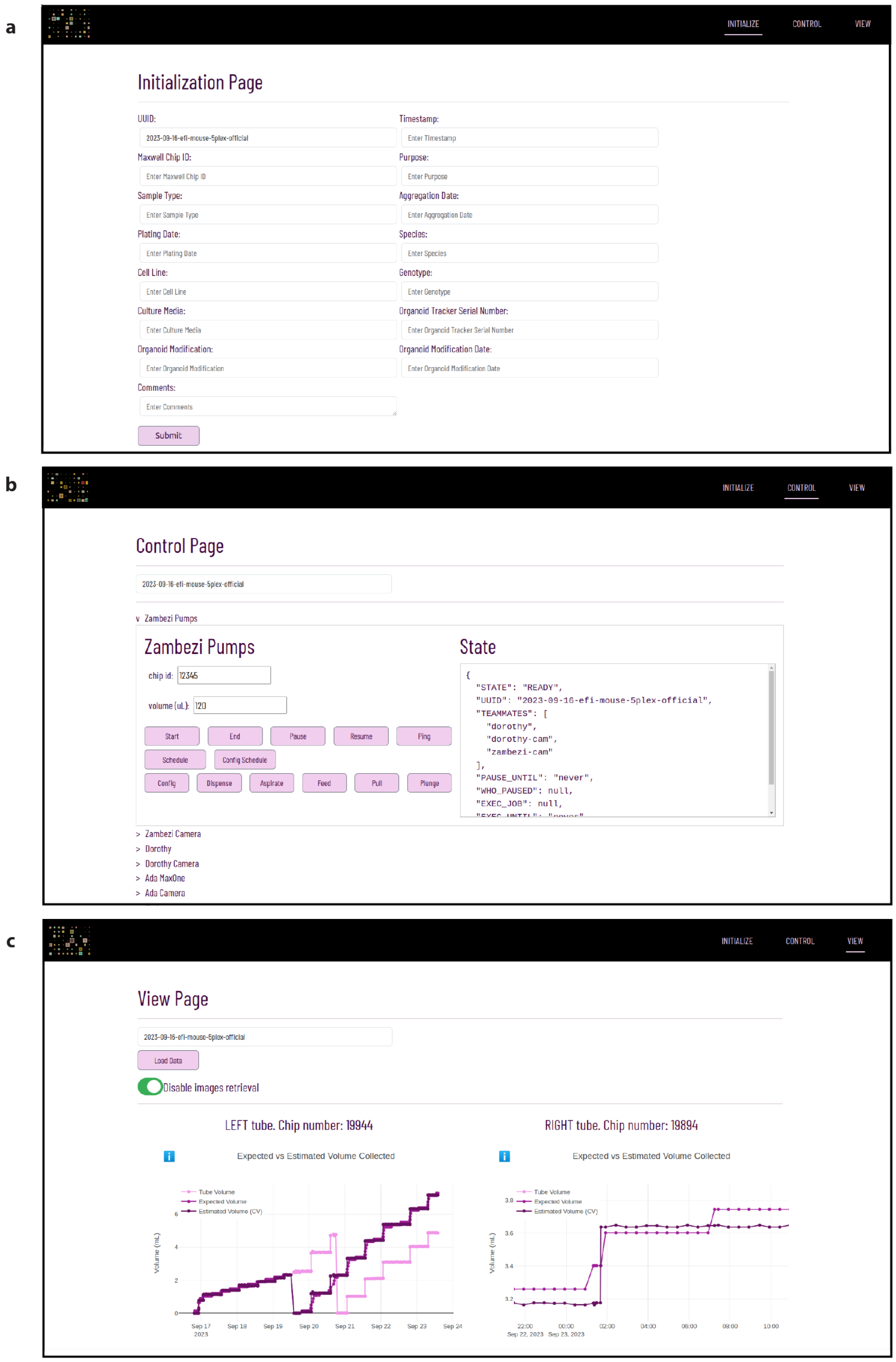

Supplementary Fig. 2: **Webpage user interface screenshots.** **(a)** Initialization page: Users can input details about the experiment and the biological samples. **(b)** Control page: Users can access and control every device involved in the experiment. **(c)** Visualization page: It includes three graph types. (1) Expected versus Estimated Volume Graph: compare volumes determined by the computer vision algorithm with volume according to pump metrics, highlighting any discrepancies and mismatching data. (2) Expected minus Estimated Graph: It shows the difference between the pump metrics and computer vision estimates for each device. They are designed to quickly identify alignment or discrepancies between these two methods, where values close to zero suggest good alignment, and deviations indicate measurement inaccuracies. (3) Collected Volume According to Computer Vision and Pump Graph: This graph contrasts the volume of media collected as reported by the pump system with that detected by the Computer Vision algorithm, which is crucial for assessing feeding accuracy. For example, if the pump indicates a feed of 300 µL, but the Computer Vision only detects 150 µL, this discrepancy is highlighted.

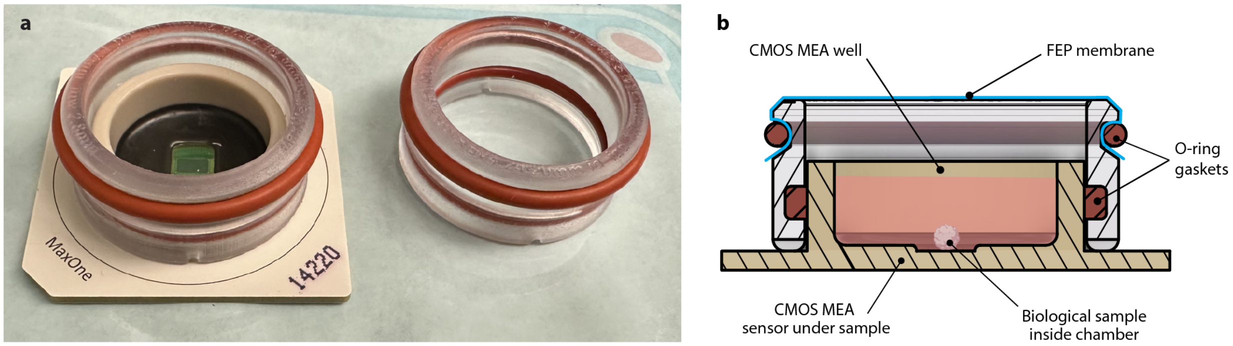

Supplementary Fig. 3: **3D printed breathable membrane lid used for Controls modeled after designs by Potter [52].** **(a)** Picture of the membrane lid and HD-MEA. The chamber is comprised of biocompatible 3D-printed parts, sealed by O-rings to the HD-MEA, and imaged through the FEP membrane stretched over the top with an O-ring. **(b)** Cross-sectional rendering depicting the fluid path and position of the sample.

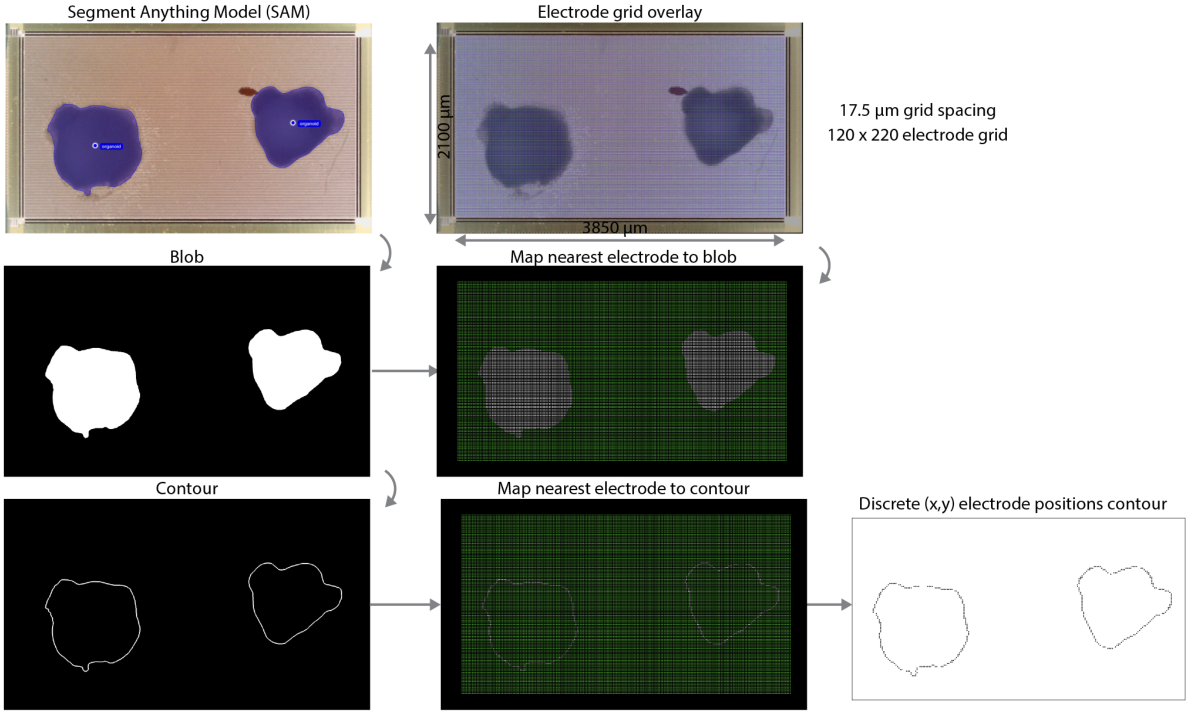

Supplementary Fig. 4: Organoid boundary segmentation process.

.

State	Description
**SHUTDOWN**	The device has been turned off gracefully and won't respond until it's turned back on.
**IDLE**	The device is not assigned to any experiments and not doing anything at the moment, and is missing physical prerequisites (i.e., a reagent or piece of hardware) to be able to perform its job.
**PRIMED**	The device is not assigned to any experiments and not doing anything at the moment, but it has all the physical prerequisites to perform its job.
**READY**	The device is assigned to an experiment and is ready to execute a command.
**WAITING**	The device has received a command to PAUSE and is waiting until a given time to resume performing jobs.
**EXEC**	The device is actively executing a job command.

Supplementary Table. 1: **Device states.** The *device-class* is structured as a finite-state machine, with a defined set of states (SHUTDOWN, IDLE, PRIMED, READY, PAUSED, EXEC) that describe its status. The finite-state machine reads a set of inputs and changes to a different state based on those inputs. The inputs can be user physical interactions (i.e., button press, linkage of consumables, etc.), MQTT messages containing job requests, or scheduled events.

Command	Description
START*	Initiates an experiment process on a specified device. The device must not be engaged in another experiment and should be in a PRIMED state. If successful, the device acknowledges the request and changes its state to READY.
END†	Ends an ongoing experiment on a device or all devices associated with an experiment UUID. The device(s) will drop current tasks and reset to the IDLE state.
STATUS†	Retrieves the current status, state, associated experiment UUID, teammates, and job schedule. Works in any device state.
PAUSE†	Temporarily halts the device's ability to start working on new commands for a specified duration. If the device is already paused or not part of an experiment, it will return an error. Otherwise, the device will successfully change its state to WAITING.
RESUME†	Requests the device to continue execution after a pause. Only the device that initiated the pause can send a resume command. The PAUSED device will successfully change its state to WAITING.
SCHEDULE†	Adds, clears, or retrieves scheduled tasks for the device. The device will execute the specified task payload at the specified time every X hours or minutes (unless it's WAITING, then it will do backlogged tasks at the easiest convenience). Works in any state.
STOP†	Requests the cancellation of a running task. If there is no task running, an error message is returned.
PING†	Requests the device to respond with a ping message. This is used to check if the device is online and listening to a given topic. Works in any state.
SLACK‡	Posts a message to Slack. The message can contain text and/or an image.

Supplementary Table. 1: **Generic commands.** The parent *device-class* responds to a generic set of commands. Commands are sent on hierarchical MQTT topics that allow widening and narrowing of scope. We used each experiment's Universal Unique Identifier (UUID) and each device's name as part of the topic. If a device is not part of an experiment, the default UUID is NONE.
* Use MQTT topic: `NONE/device` because no experiment assigned yet
† Use MQTT topic: `UUID/device` or just `UUID` to address all
‡ Use MQTT topic: `TOSLACK`

**Command**	**Device**	**Description**
RECORD	HD-MEA	Performs an electrophysiology recording for a defined period of time.
PICTURE	Camera(s)	Takes a picture from the camera.
FEED	Pump	Performs a cycle of aspirating spent and dispensing fresh media of the pre-configured volume.
ASPIRATE	Pump	Aspirates a specified volume of liquid (mL) from the culture chamber.
DISPENSE	Pump	Dispenses a specified volume of liquid (mL) to the culture chamber.
PULL	Pump	A rapid, full-syringe aspiration to assist pulling media through high resistance or clogs.
SPIKESORT	Spike sorting	Spike sorts a specified dataset stored in S3 using the analysis pipeline.
ESTIMATE	Estimator	Estimates the amount (mL) of media in a collection reservoir by applying computer vision analysis to a specified image of the collection reservoir stored in S3.

Supplementary Table. 1: **Application-specific commands.** The child *device-classes* extend the top level *device-class*, respond to all genetic commands as well as their instrument-specific commands. New commands can be easily defined and implemented for a specific experimental application by extending *device-class* child. For all commands above, use MQTT topic: `UUID/device_name`.
