# Supplementary figures and images for: A feedback-driven brain organoid platform enables automated maintenance and high-resolution neural activity monitoring
Source: Internet Things (Amst). Author manuscript; Available in PMC 2026 Jan 9. (PMC12781996; doi:10.1016/j.iot.2025.101671)

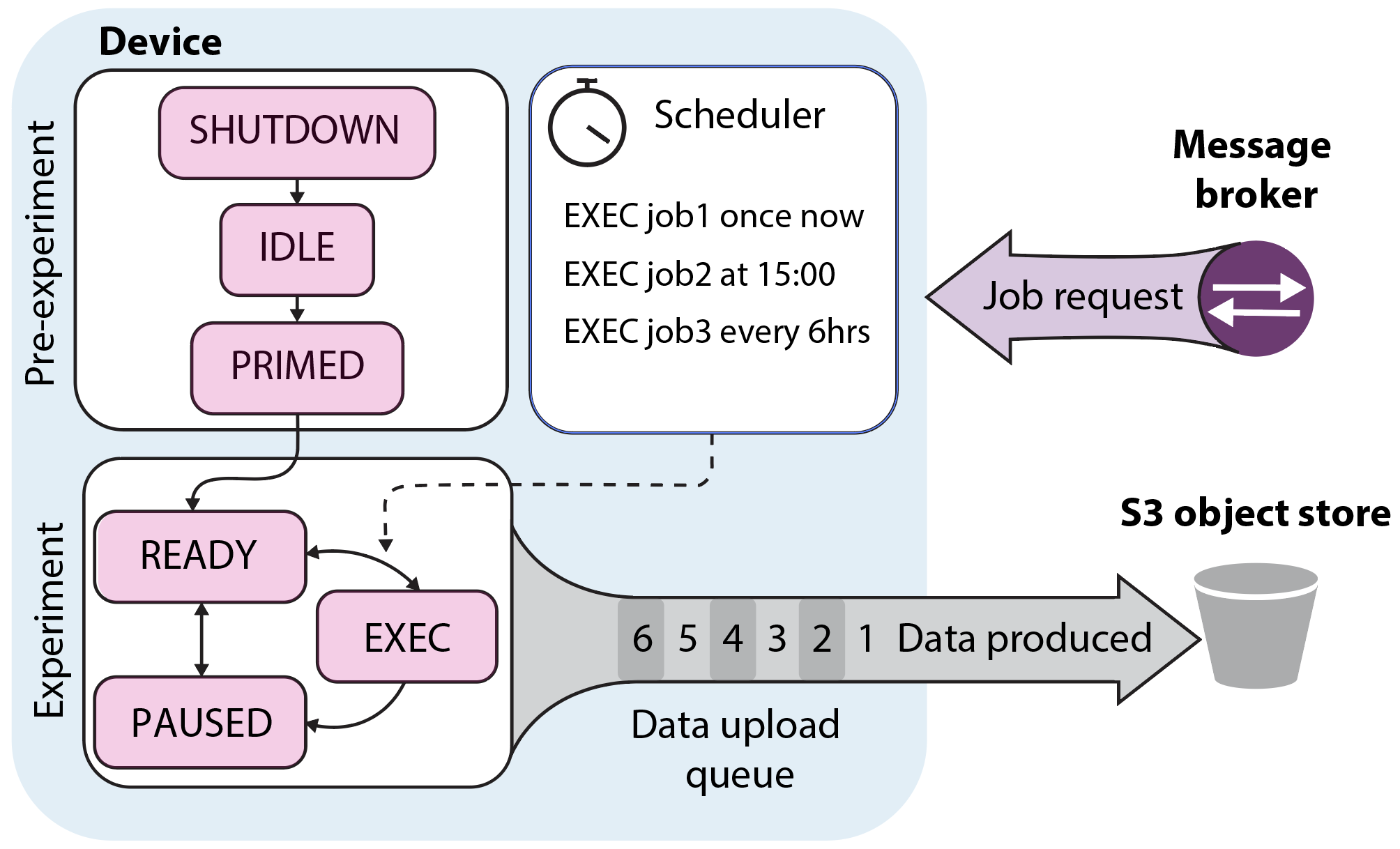

Supplement: SuppMat2 [file NIHMS2123043-supplement-SuppMat2.zip › supplemental_files_and_code/img/mqtt_device_state_machine_summary.png]

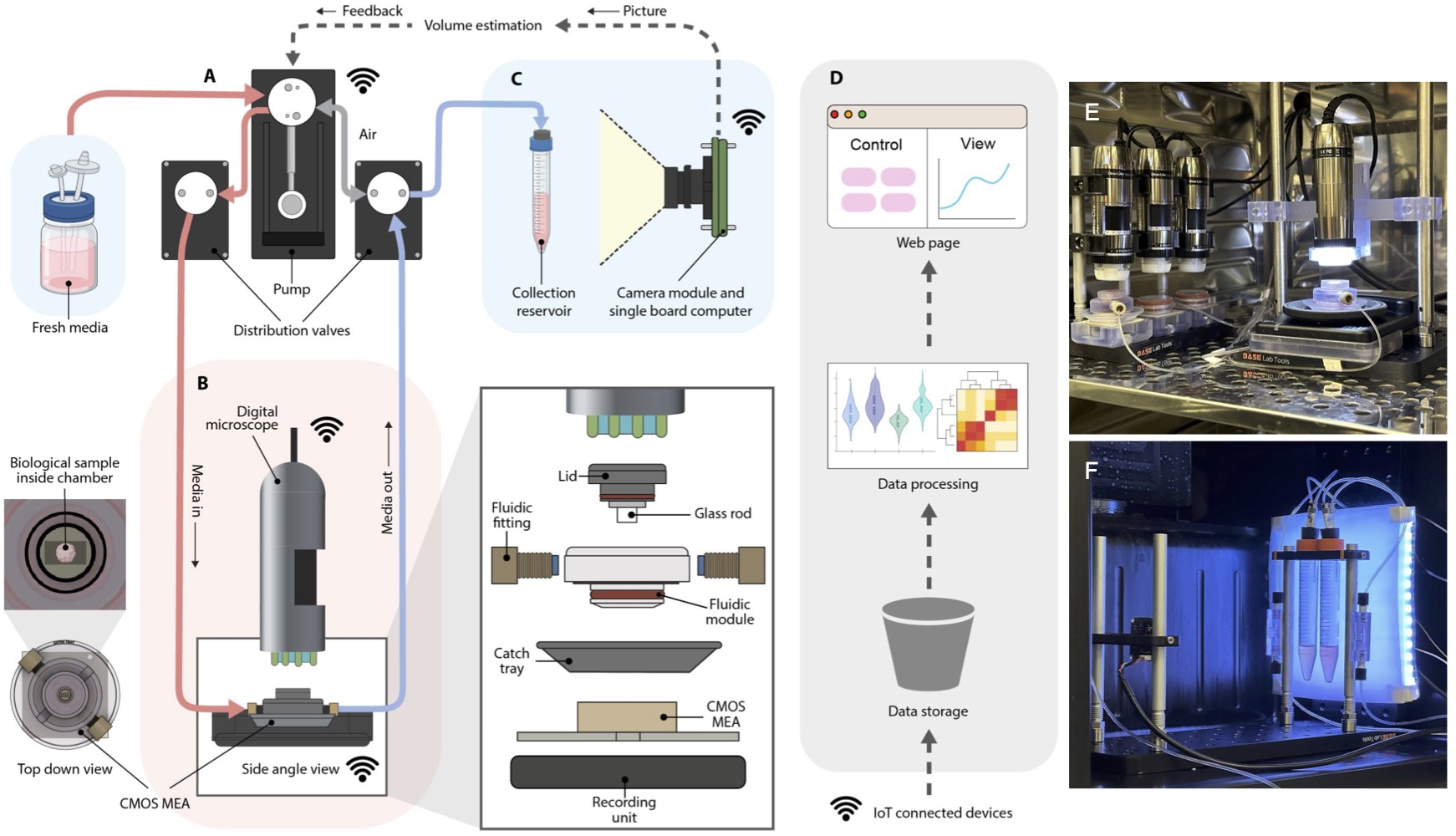

Supplement: SuppMat2 [file NIHMS2123043-supplement-SuppMat2.zip › supplemental_files_and_code/img/overview.jpg]

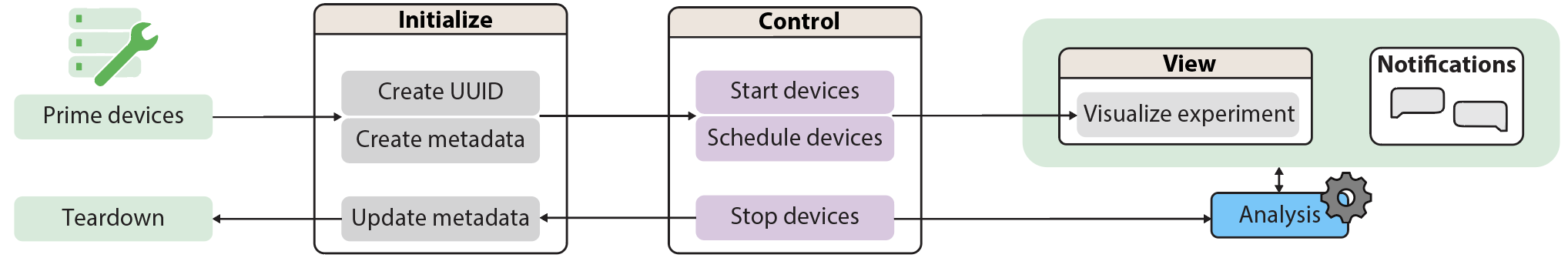

Supplement: SuppMat2 [file NIHMS2123043-supplement-SuppMat2.zip › supplemental_files_and_code/img/user-workflow.png]

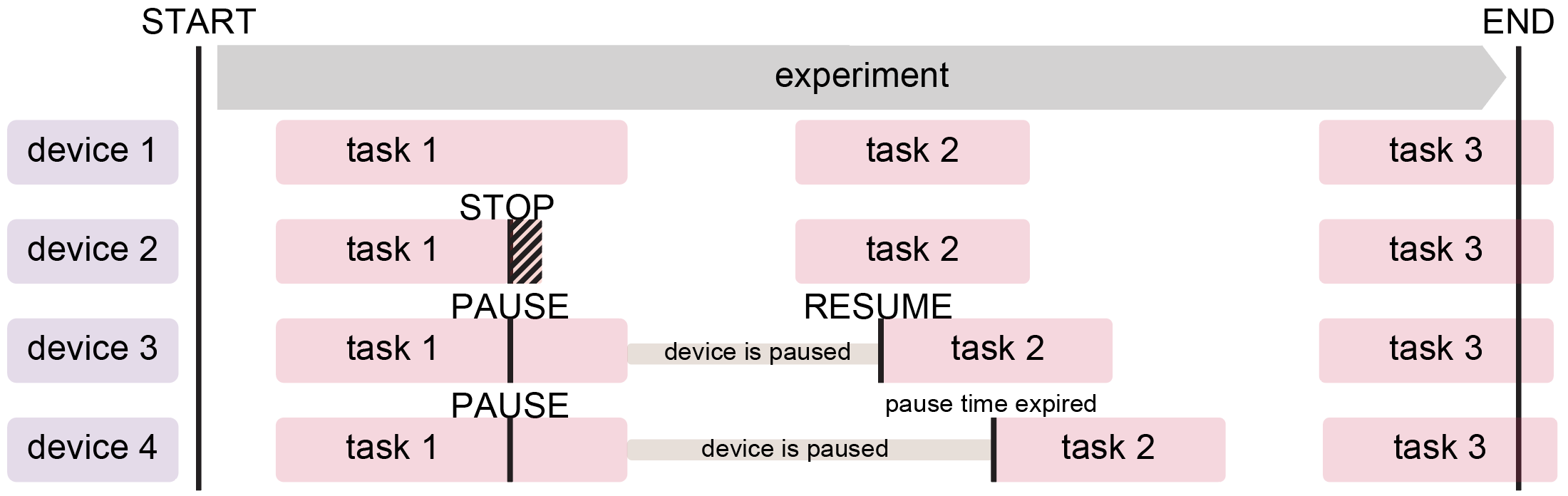

Supplement: SuppMat2 [file NIHMS2123043-supplement-SuppMat2.zip › supplemental_files_and_code/img/cmd_visu.png]
